# Engineered Peptide Coacervates Enable Efficient Intracellular Delivery of the MYC Inhibitor omoMYC

**DOI:** 10.1021/acs.molpharmaceut.5c00468

**Published:** 2025-04-30

**Authors:** Carmine P. Cerrato, Martin Krkoška, Yue Sun, Judit Liaño-Pons, Qi Ying Neo, Thibault Vosselman, Mohammad Alzrigat, Borek Vojtěšek, David P. Lane, Marie Arsenian Henriksson, Ali Miserez, Michael Landreh

**Affiliations:** † Department of Microbiology, Tumor and Cell Biology, Karolinska Institutet, 171 65 Stockholm, Sweden; ‡ Research Centre for Applied Molecular Oncology, Masaryk Memorial Cancer Institute, 656 53 Brno, Czech Republic; § Centre for Sustainable Materials, School of Materials Science and Engineering, Nanyang Technological University (NTU), 50 Nanyang Avenue, Singapore 637553, Singapore; ∥ School of Biological Sciences, Nanyang Technological University (NTU), 60 Nanyang Drive, Singapore 637551, Singapore; ⊥ Department of Cell and Molecular Biology, Uppsala University, 751 24 Uppsala, Sweden

**Keywords:** drug delivery, liquid−liquid phase separation, protein engineering

## Abstract

Intracellular delivery is a bottleneck in the development of therapeutic peptides and proteins. Here, we demonstrate the efficient delivery of omoMYC, the first MYC inhibitor in clinical trials, using HB*pep-SP*, an engineered peptide forming liquid–liquid phase-separated coacervates. HB*pep*-SP coacervates facilitate efficient cellular uptake and intracellular delivery of the omoMYC peptide at concentrations lower than those required for spontaneous uptake. Strikingly, omoMYC coacervates result in reduced proliferation and apoptosis induction in the low c-MYC expressing cell lines HEK293 and SH-SY5Y cells, but not in HeLa and SK-N-BE(2) cells with high c-MYC/MYCN expression, respectively, suggesting that endogenous MYC/N levels may impact the effects of omoMYC. Importantly, our approach bypasses the need for cell penetration-enhancing chemical modifications, offering a novel strategy for the investigation of peptide drug mechanisms in therapeutic development.

## Graphical abstract

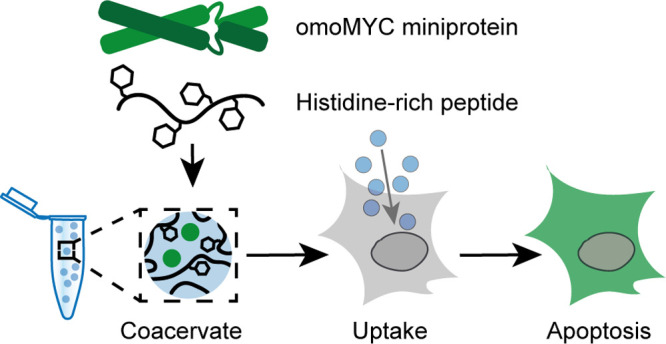

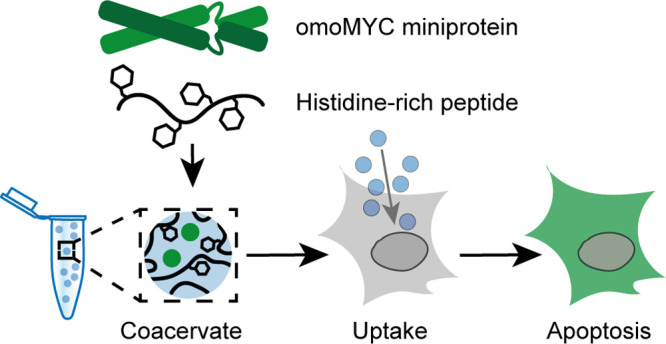

## Introduction

Transcription factors (TFs) are proteins that play crucial roles in gene regulation and generally lack a well-defined, stable structure.^1^ They often contain intrinsically disordered regions that allow interactions with a wide variety of partners.^2^ For example, many TFs can participate in liquid–liquid phase separation (LLPS), providing a flexible way to assemble the transcription machinery in a dynamic manner to control gene expression.^3^ Disordered TFs are key players in processes related to cell division and cell growth and thus potential targets for cancer therapy. However, flexibility is a hurdle for structure-based drug development, as the proteins lack well-defined binding pockets that can be targeted with small molecules.^4^

The MYC proto-oncoproteins are prominent examples of disordered TFs whose high potential for cancer therapy has been hampered by their dynamic structures.^4^ They are major drivers of cell growth and are found dysregulated in around >70% of all human cancers.^5–7^ The C-terminus of MYC contains a basic helix-loop-helix (bHLH) domain with a leucine zipper that forms a stably folded dimer with MAX and binds to DNA E-boxes with nanomolar affinity.^8–10^ Based on the MYC-MAX structure, Soucek and colleagues developed omoMYC, a homodimeric form of the MYC bHLH domain containing four amino acid mutations designed to inhibit MYC activity by preferentially binding to MAX and omoMYC itself and partially to MYC; these dimers thus inhibit MYC function by blocking MYC-MAX binding.^11^ omoMYC has recently shown promise in Phase I clinical studies, demonstrating tolerability and stable disease in several of the patients as well as the ability to interfere with the MYC driven transcriptional programs.^12^ This peptide thus represents the most advanced therapeutic approach for specifically targeting MYC-dependent cancers to date.^13^

The ability to cross the cell membrane is an important feature of any viable drug candidate. While small molecules may cross the membrane with the help of membrane channels or transporters, larger therapeutics, such as peptide drugs, require intrinsic cell-penetrating ability to enable efficient target engagement in the cell. This hurdle is increasingly difficult to overcome, the more complex a peptide-based molecule becomes. omoMYC is a cell-penetrating peptide (CPP), whose ability to cross the cell membrane is mediated by amphipathic helices. However, spontaneous uptake of the covalently linked omoMYC dimer is relatively inefficient, likely due to its large size of 26 kDa.^14–16^ Chemical modifications have been employed to improve internalization, including conjugation to a CPP sequence, or shortening of the helices that stabilizes its dimeric structure by forming a leucine zipper.^15,16^ Here, we explore an alternative way to deliver unmodified omoMYC using HB*pep*-SP, an engineered histidine-rich peptide derived from a squid beak protein, which spontaneously forms coacervates via pH-triggered LLPS.^17,18^ HB*pep*-SP coacervates can cross the cell membrane through a mechanism sharing features of both macropinocytosis and phagocytosis.^19^ In HB*pep*-SP, its single lysine is conjugated with a disulfide bond-containing self-immolative moiety that triggers disassembly of the liquid-like droplets under the reducing conditions it encounters in the cytosol, as evidenced by the fact that release can be tuned by modulating intracellular glutathione levels.^20–22^ Cargo molecules that are present during LLPS assembly of the peptide are instantaneously recruited within the coacervates and are subsequently efficiently released upon disassembly of the coacervates occurring in the cytosol (Figure 1a).^20^ The system can be optimized for other cargos such as mRNAs by modifying the peptide sequence.^21^

**Figure 1:**
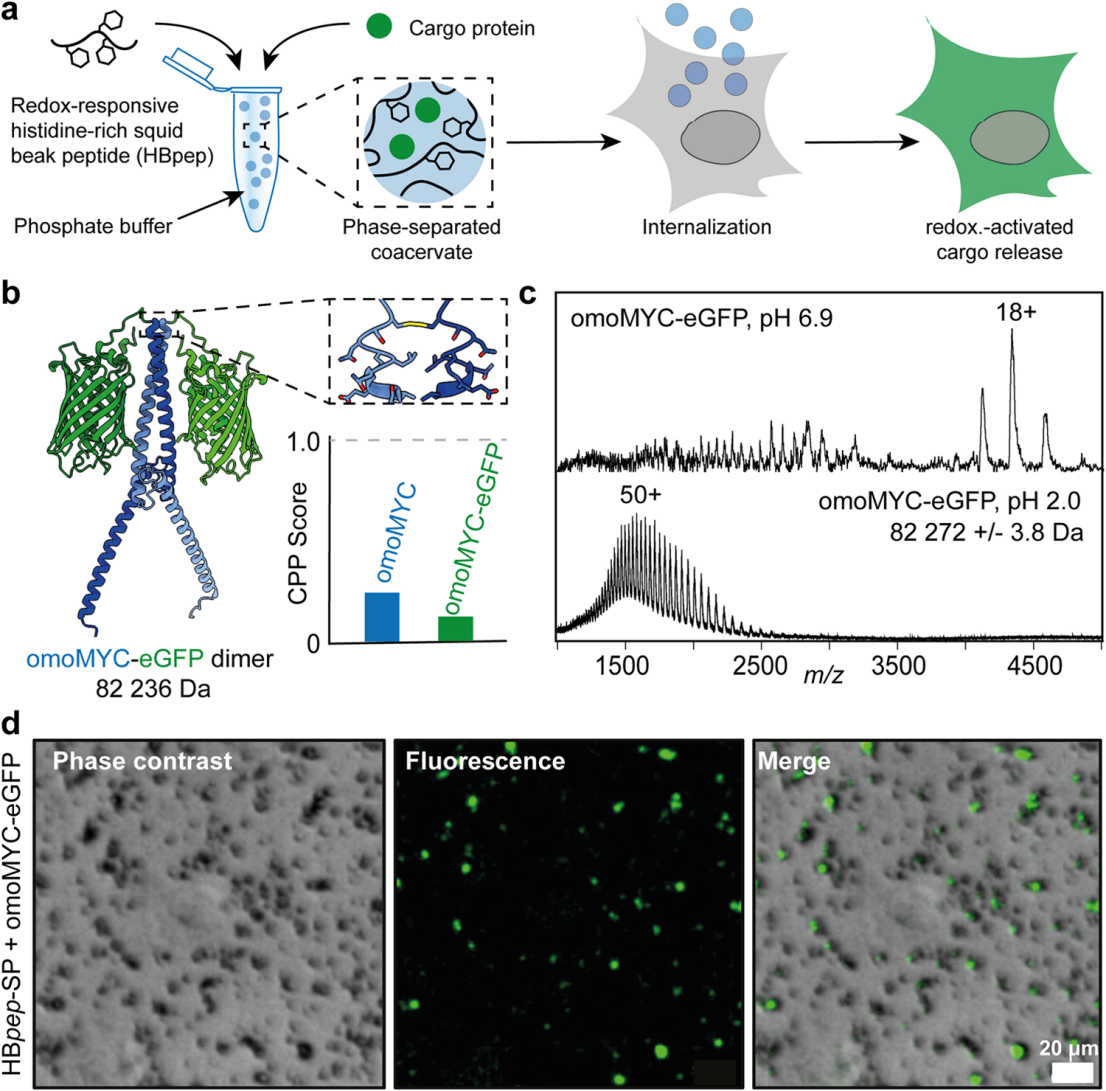
Principle and characterization of omoMYC-eGFP/HB*pep*-SP coacervates. (a) Principle of HB*pep*-SP/cargo coacervate assembly and intracellular delivery. (b) Alphafold3 generated the structure of the engineered omoMYC-eGFP dimer, with omoMYC shown in blue and green fluorescent protein (GFP) in green. The protomers are linked by a disulfide bond between Cys 90 on the omoMYC C-terminal side. Inset: Cell penetration probability (CPP) scores from C2Pred (http://lin-group.cn/server/C2Pred) indicate that omoMYC and omoMYC-eGFP have a low potential to cross the cell membrane. (c) Mass spectrometric analysis of recombinant omoMYC-eGFP shows a compact dimer at pH 6.9 (top). Acid-induced denaturation does not dissociate the dimer (bottom). (d) Incubation of omoMYC-eGFP with HB*pep*-SP results in the formation of GFP-positive coacervates in HEK293 cells. Scale bar: 20 μm.

## Materials and Methods

The full materials and methods are given in the Supporting Information.

## Results

### HB*pep*-SP Enables Efficient Delivery of a Non-Cell-Penetrating omoMYC Variant

To be able to follow the encapsulation and delivery of omoMYC with HB*pep*-SP, we designed an omoMYC variant with a C-terminal eGFP moiety attached by a six-glycine linker (Figure 1b). In omoMYC, both dimer subunits are linked by a disulfide bond between the cysteines at position 90. Alphafold3 predictions of the fusion protein suggest that the GFP moiety does not affect covalent dimerization of omoMYC. Importantly, both omoMYC and omoMYC-eGFP have low probabilities of cell penetration, as predicted by the C2Pred server (Figure 1b).^23^ Next, we recombinantly produced omoMYC-eGFP in Escherichia coli and analyzed the purified protein with mass spectrometry. At neutral pH, we detected a narrow charge state distribution with an average mass of 82.2 kDa, in line with a compactly folded omoMYC-eGFP dimer. pH-induced denaturation yielded highly charged ions, indicative of complete unfolding, but no monomers, confirming covalent dimerization of omoMYC (Figure 1c). Circular dichroism (CD) spectroscopy shows a mixture of β-sheets and α-helices, in line with the predicted structure (Figure S1). Loading of the coacervates was initiated by mixing the omoMYC-eGFP cargo protein and the HB*pep*-SP peptide in phosphate buffer at pH 6.5.^20^ Confocal microscopy confirmed the formation of clusters of micrometer-sized coacervate droplets that exhibit strong green fluorescence (Figure 1d). We conclude that omoMYC-eGFP is readily encapsulated by HB*pep*-SP.

After loading the HB*pep*-SP coacervates, we tested the delivery of omoMYC into the cytosol of human embryonic kidney (HEK) 293 and cervical cancer HeLa cells. Following established protocols,^20^ we incubated the cells with either omoMYC alone or with omoMYC/HB*pep*-SP coacervates for 4 h at a cargo concentration of 0.5 μM and assessed uptake with fluorescence microscopy (Figure S2). No green fluorescence was observed in the cytosol of cells incubated with omoMYC-eGFP alone (Figure S2), in line with reports that neither omoMYC nor GFP were taken up by HEK293 and HeLa cells at similar concentrations.^15,20^ In contrast, incubation with omoMYC-eGFP coacervates resulted in a strong GFP signal in nearly all cells, indicating widespread cytosolic and nuclear distribution of the peptide. A minor population of the HeLa cells contained GFP-positive *punctae*, suggesting that the coacervates had been taken up but not yet disassembled (Figure S2).^20^

### omoMYC/HB*pep*-SP Coacervates Reduce the Viability of HEK293 But Not HeLa Cells

Having established that loaded coacervates are efficiently taken up by the cells, we interrogated whether the internalized omoMYC affects the cell viability. Since GFP can induce apoptosis in some cell types,^24^ we switched to unmodified omoMYC, which is in clinical trials as OMO103.^12^ Previous viability studies reported IC_50_ values of 5–12 μM for direct incubation of cancer cell lines with omoMYC.^14^ Similarly, addition of 1 μM omoMYC to the culture medium did not affect the viability of HEK239 or HeLa cells, likely since too little peptide is internalized at low concentrations to elicit a response.^15^ To test coacervate-mediated uptake, we chose an omoMYC concentration of 0.5 μM.^25^ Cells were treated with either cargo-free coacervates composed of HB*pep*-SP or omoMYC alone, or with omoMYC/HB*pep*-SP coacervates for 24 h, with 4 h under serum-free conditions, in line with the use of low-serum or serum-free conditions in previous omoMYC uptake studies.^15^ Viability was assessed with the PrestoBlue assay after 24 h of treatment, as well as 2 and 6 days after peptide removal. We observed significantly reduced viability in HEK293 cells following 24 h of incubation with omoMYC/HB*pep*-SP coacervates compared to omoMYC or HB*pep*-SP alone (Figure 2a), while HeLa cells did not show significant changes in viability at any time point (Figure 2b). To assess whether the reduction in viability of HEK293 cells may be related to membrane damage from the coacervates, we monitored the release of lactate dehydrogenase (LDH).^26^ However, no differences in LDH levels in medium were detected between the conditions, suggesting that cell membranes remain intact (Figure S3). Together, our observations suggest that omoMYC/HB*pep*-SP coacervates, but not HB*pep*-SP or omoMYC alone, can induce cell death in HEK293 cells without any cell membrane damage.^27^

**Figure 2:**
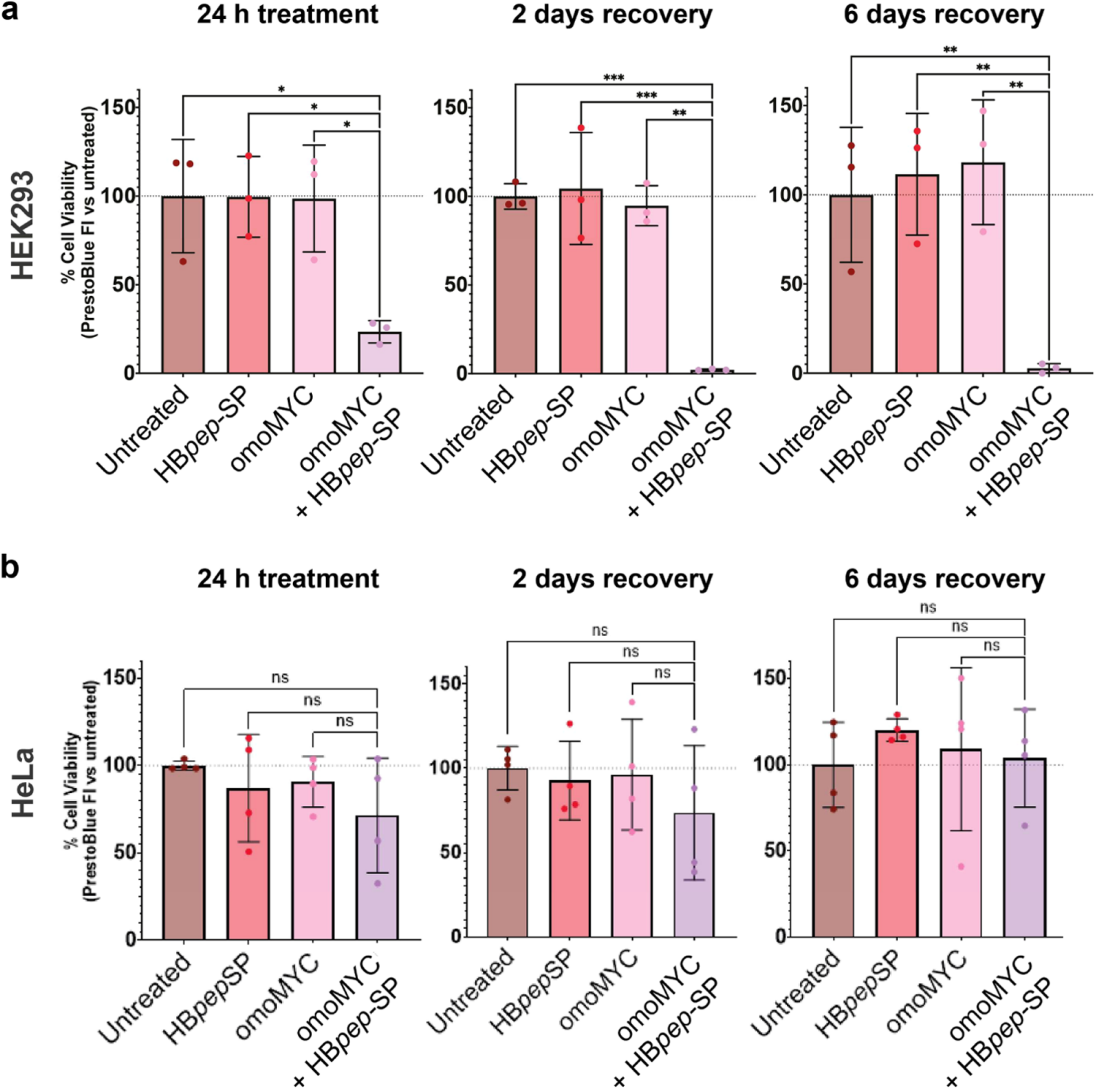
Effect of omoMYC/HB*pep*-SP coacervates on the viability of HEK293 and HeLa cells. (a) HEK293 cells and (b) HeLa cells were analyzed with the PrestoBlue assay following 24 h treatment, as well as after 24 h treatment and 2 or 6 days after peptide removal. At all time points analyzed, HEK293 cells (top row) showed significantly reduced viability in the PrestoBlue assay when treated with omoMYC/HB*pep*-SP coacervates compared to treatment with omoMYC or HB*pep*-SP alone. In contrast, there were no significant changes in viability in HeLa cells in response to any of the treatments except for a small reduction after 2 days of recovery by omoMYC/HB*pep*-SP coacervates. Data are presented as mean ± standard deviation (SD), *n* = 3 biological independent replicates for HEK293 cells, and *n* = 4 biological independent replicates for HeLa cells. Statistical significance was determined by RM one-way analysis of variance (ANOVA), with the Geisser-Greenhouse correction, Tukey’s multiple comparisons test, with individual variances computed for each comparison. Statistical significance: *p < 0.05, **p < 0.01, ***p < 0.001.

### omoMYC/HB*pep*-SP Coacervates Induce Apoptosis in HEK293 But Not in HeLa Cells

Next, we aimed to elucidate the potential mechanisms underlying the action of the omoMYC/HB*pep*-SP coacervate treatment. For this purpose, we focused on the simultaneous analysis of cell cycle distribution as well as apoptosis and DNA damage markers using flow cytometry. We monitored cell cycle alterations, DNA damage activation, and induction of apoptosis in response to treatment after 24 h as well as after 2 days of recovery. Cell cycle analysis showed that HEK293 cells progressed into the G2/M phase, accompanied by an around 30% increase in the number of sub-G1 phase cells following incubation with omoMYC/HB*pep*-SP coacervates (Figures 3 and S4). After 24 h of treatment and after 2 days of recovery with either HB*pep*-SP or omoMYC alone, only few cells remained in the omoMYC/HB*pep*-SP-treated group (Figures S4 and S5). This suggests either enhanced susceptibility to apoptosis and/or deactivation of cell cycle checkpoints that could lead to mitotic catastrophe. Fluorescence-activated cell sorting (FACS) analysis further showed that HEK293 cells exhibited significantly enhanced caspase-3 (Figures 3a,b, S4, and S5) and poly­(ADP-ribose) polymerase (PARP) (Figures 3a,b and S4) cleavage, hallmarks of apoptosis.^28^ In contrast, we did not detect any change in histone H2A.X phosphorylation in line with reports that omoMYC does not induce DNA damage.^29^ Notably, no significant changes in cell cycle distribution were observed in HeLa cells (Figures 3, S4, and S5). These findings rationalize the decreased viability in HEK293 cells following treatment with omoMYC-loaded coacervates, indicating increased apoptosis (Figure S4). Notably, at the 24 h time point, HeLa cells showed a significant increase (around 15%) in sub-G1 cells as well as PARP cleavage (around 12%), indicating that apoptotic pathways are also activated in these cells after incubation with omoMYC-loaded coacervates.

**Figure 3:**
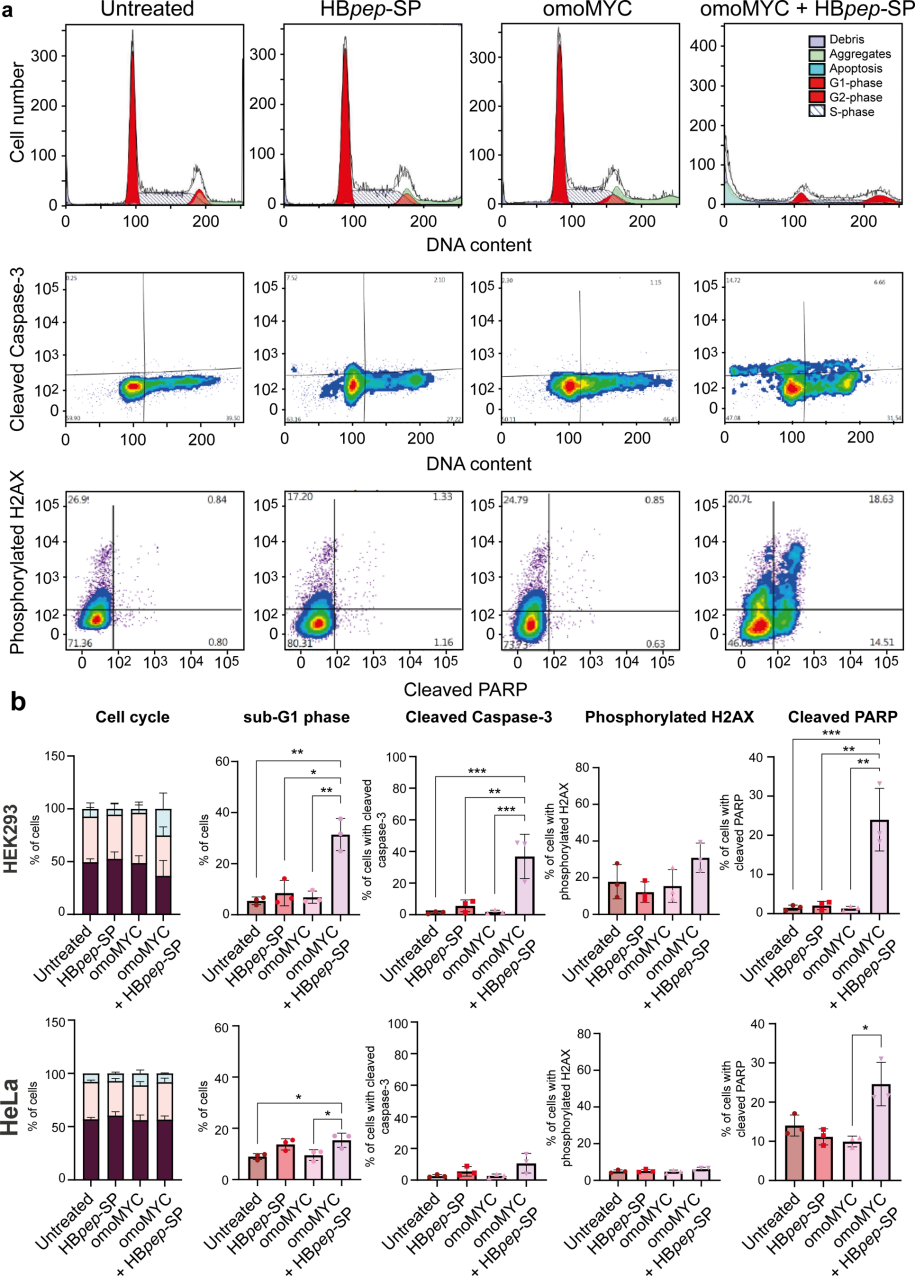
omoMYC/HB*pep*-SP coacervates induce apoptosis in HEK293 but not HeLa cells. (a) Flow cytometry plots for HEK293 cells after 24 h treatment, showing cell cycle distribution (top row), caspase-3 cleavage (middle row), and H2A.X phosphorylation (bottom row). (b) Quantification of the FACS analysis of HEK293 (top row) and HeLa (bottom row) cells following 24 h of incubation with omoMYC/HB*pep*-SP coacervates. Neither omoMYC nor HB*pep*-SP alone significantly affected the cell cycle in either cell line. omoMYC coacervates induced a significant increase in cell death as well as increased PARP cleavage (fifth column), while too few cells remained for reliable cell cycle analysis following combined treatment. HB*pep*-SP or omoMYC alone did not induce significant changes in any of the parameters compared to untreated control HEK293 or HeLa cells but the latter cells showed increased cleaved PARP following combination treatment. Furthermore, neither HEK293 nor HeLa cells showed a significant increase in H2A.X phosphorylation. Data are presented as mean ± SD, *n* = 3 biological independent replicates. Statistical significance was determined by one-way ANOVA, Tukey’s multiple comparisons test, with a single pooled variance. Statistical significance: *p < 0.05, **p < 0.01, ***p < 0.001.

### omoMYC/HB*pep*-SP Coacervates Induce Apoptosis-Related Morphological Changes in HEK293 Cells

In parallel, we analyzed the activity of omoMYC/HB*pep*-SP coacervates in HEK293 and HeLa cells by immunofluorescence and confocal microscopy using markers for cell death induction and DNA damage, respectively. In addition, we assessed whether omoMYC/HB*pep*-SP caused changes in the cell morphology using phalloidin staining. We observed strong induction of caspase-3 cleavage and rounding of the cells, indicating apoptosis, after omoMYC/HB*pep-*SP coacervate incubation, with only basal levels of apoptosis, DNA damage markers, and no pronounced morphological changes after treatment with the coacervate peptide HB*pep*-SP alone (Figures 4, S6, and S7). Notably, these results are consistent with the observed cell cycle analysis (Figure 3) and align with previous observations that omoMYC can enhance MYC-induced apoptosis in myoblasts and glioma cells.^30,31^ As expected, the coacervate-mediated delivery of omoMYC, but not HB*pep*-SP alone, significantly inhibited cell proliferation as evidenced by the reduction of cell number in HEK293 cells at all time points analyzed (Figures 3 and S5). In HeLa cells, this effect was most pronounced after 24 h of incubation and persisted for 2 days and was almost completely reversed after 6 days of recovery. HB*pep*-SP alone had minimal effects on cell morphology compared to untreated cells (Figures 4, S6, and S7). As for cell morphology, no significant changes were observed in HEK293, while HeLa cells appeared elongated upon incubation with omoMYC-HB*pep*-SP compared to untreated or coacervates alone.

**Figure 4:**
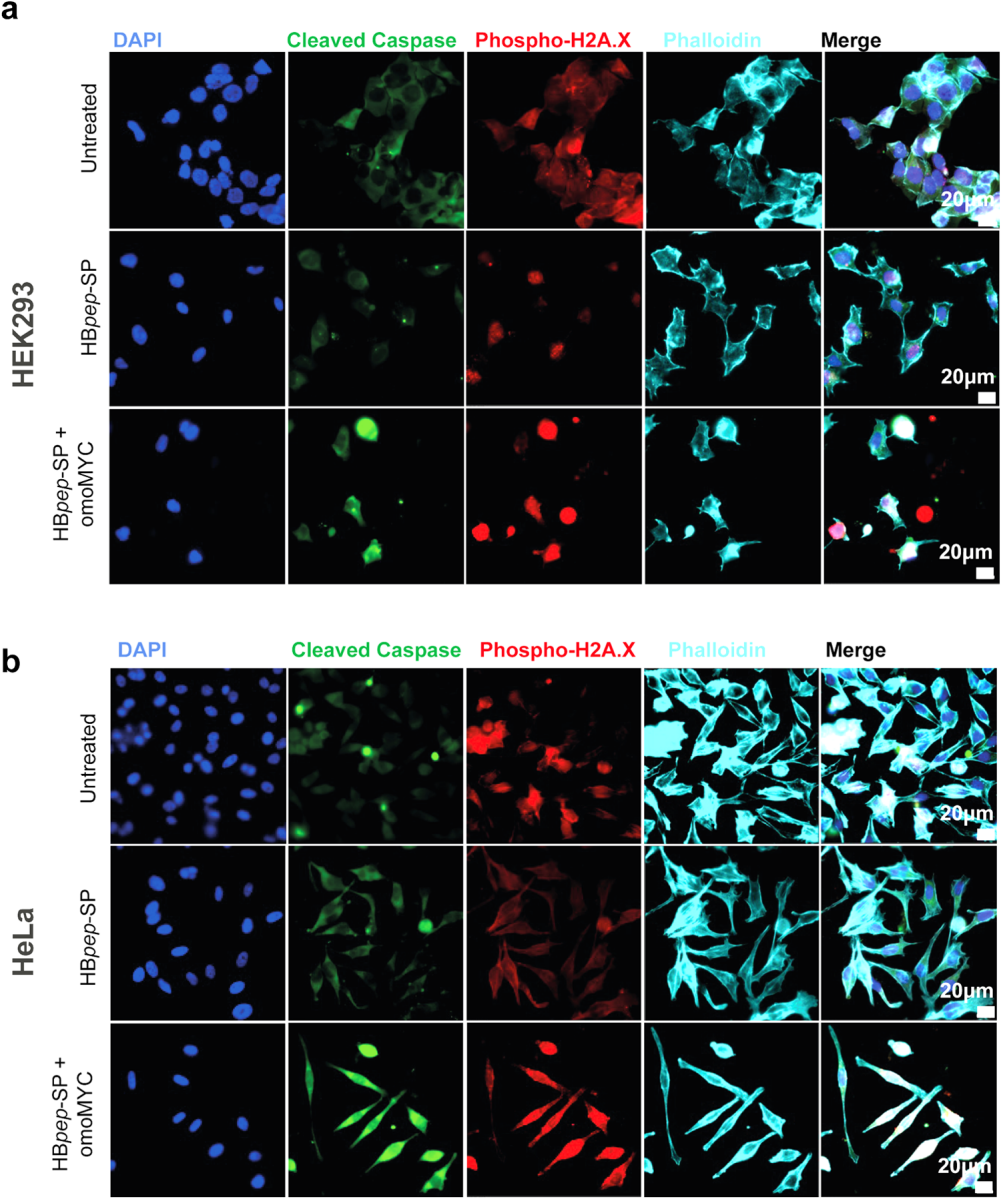
Apoptosis activation induced by omoMYC/HB*pep*-SP coacervates in HEK293 and HeLa cells. Representative confocal microscopy images of (a) HEK293 and (b) HeLa cells after 24 h of treatment with omoMYC/HB*pep*-SP coacervates. Nuclei (blue); cleaved caspase-3 (green), H2A.X phosphorylation (red), and phalloidin (turquoise) were used as markers to detect apoptotic cell death, DNA damage, and F-actin, respectively. Merged images show changes in cell confluence/morphology and marker distribution. Confocal microscopy images were acquired using 395/488/555/640 nm laser lines. Representative images from three biologically independent experiments are presented (*n* = 3). Scale bars: 20 μm.

### omoMYC/HB*pep*-SP Coacervate Treatment Elicits Distinct Effects in Two MYC-Driven Neuroblastoma Cell Lines

Having established that omoMYC/HB*pep*-SP coacervates specifically induce cell death in HEK293 while not in HeLa cells, we asked whether the approach would allow us to detect differences in the apoptotic induction of omoMYC also in neuroblastoma cell lines established from metastatic bone marrow tumors from stage 4 patients. Since omoMYC targets both c-MYC and MYCN proteins,^33^ we used SH-SY5Y cells which express c-MYC, and SK-N-BE(2) cells which carry a *MYCN*-amplification absent in SH-SY5Y cells. We analyzed the levels of c-MYC and MYCN in all four cell lines using Western blot and verified their c-MYC/MYCN expression (Figures 5 and S8). Notably, in line with previous reports, we found that HeLa cells express high c-MYC levels.^32^

**Figure 5:**
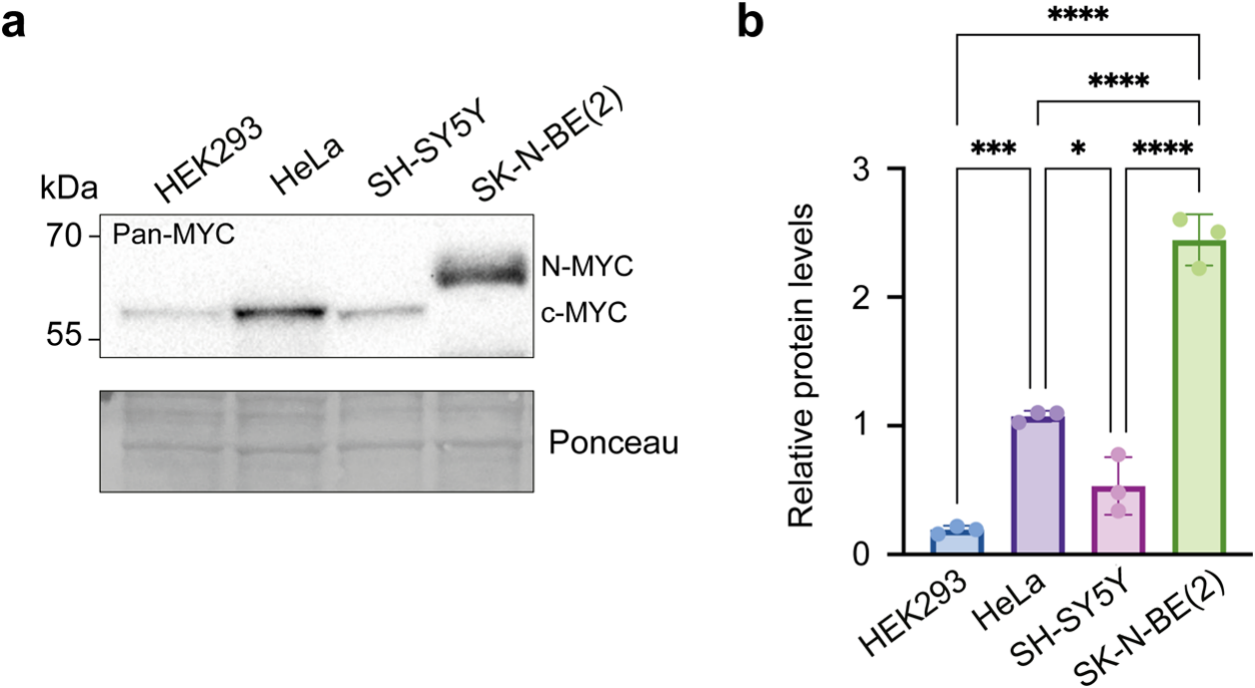
Western blot of endogenous MYC levels in HEK293, HeLa, SH-SY5Y, and SK-N-BE(2) cells. (a) Representative Western blot from four independent biological experiments stained with a pan-MYC antibody recognizing both c-MYC and MYCN. Ponceau staining was used as protein loading control. Molecular weight markers in kDa are indicated on the left. (b) Quantification of the MYC levels (*n* = 4) normalized to the Ponceau signal. One-way ANOVA multiple comparisons test, statistical significance: *p < 0.05, **p < 0.01, ***p < 0.001, ****p < 0.0001.

Cells were subjected to a similar treatment strategy as for HEK293 and HeLa cells, with 24 h treatment with or without 2 or 6 days of recovery. Internalization of omoMYC was confirmed using confocal microscopy of cells incubated with omoMYC-eGFP, as well as immunofluorescence with an omoMYC-specific antibody (Figure 6). We observed a clear increase in intracellular omoMYC-eGFP when cells were treated with omoMYC-eGFP/HB*pep*-SP coacervates compared with omoMYC-eGFP alone. Confocal microscopy of cells treated with omoMYC with and without HB*pep*-SP showed a similar uptake pattern when stained with the anti-omoMYC antibody (Figure S9). As in HEK293 and HeLa cells, no significant increase in LDH release was detected under any of the conditions in the neuroblastoma cells (Figure S10). When assessing the viability with the PrestoBlue assay, SH-SY5Y cells showed significantly reduced viability upon 24 h treatment with omoMYC/HB*pep*-SP coacervates, followed by either 2 or 6 days of recovery. For SK-N-BE(2) cells, we did not observe any reduced viability compared to the untreated control (Figure 6).

**Figure 6:**
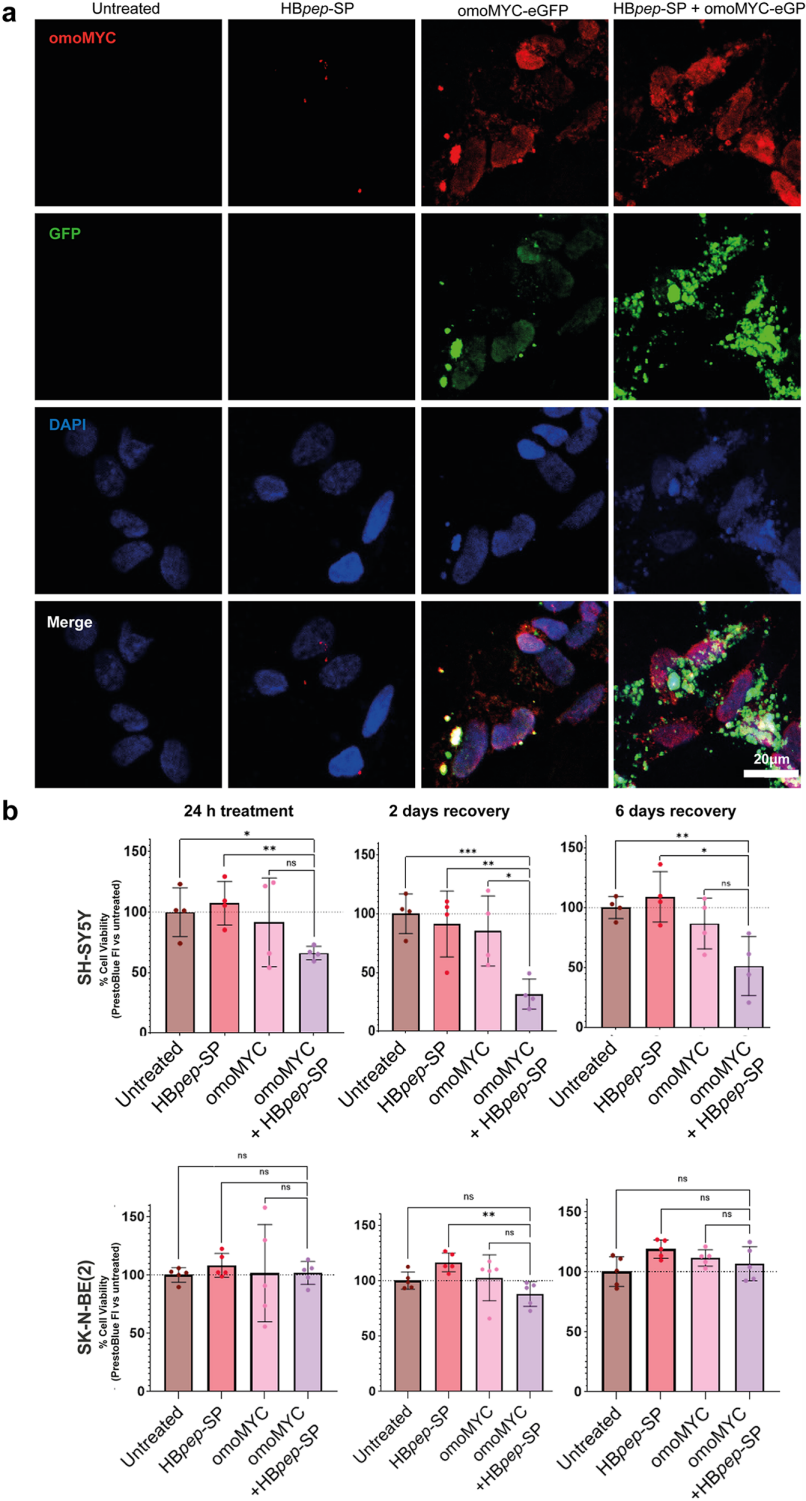
Effect of omoMYC delivery with HB*pep*-SP on the viability of SH-SY5Y and SK-N-BE(2) cells. (a) Confocal microscopy of SH-SY5Y cells treated with 0.5 μM omoMYC-eGFP (green) with or without HB*pep*-SP and stained with DAPI (blue) and the anti-omoMYC antibody (red) shows coacervate-mediated omoMYC uptake. Images were acquired using 395, 488, 555, and 640 nm laser lines. Scale bar: 20 μm. (b) Cells were analyzed for viability with the PrestoBlue assay after 24 h treatment and following 24 h treatment with or without 2 or 6 days of recovery. Data are presented as mean ± SD, *n* = 4 biological independent replicates for SH-SY5Y and *n* = 5 biological independent replicates for SK-N-BE(2) cells. Statistical significance was determined by RM one-way ANOVA, with the Geisser-Greenhouse correction, Tukey’s multiple comparisons test, with individual variances computed for each comparison. Statistical significance: *p < 0.05, **p < 0.01.

Next, we used FACS to monitor effects on cell cycle progression and induction of apoptosis after 24 h treatment as well as 2 days of recovery with the same panel of markers as for HeLa and HEK293 cells (see Figure 3). Treatment with either omoMYC or HB*pep*-SP had no significant effect in either of the neuroblastoma cell lines compared to the control. In contrast, as in HEK293 cells, few SH-SY5Y cells remained after 24 h of omoMYC/HB*pep*-SP treatment or after 2 days of recovery. Notably, cell cycle progression of SK-N-BE(2) cells remained unaffected at both time points (Figures 7 and S11). Analysis of caspase-3 and PARP cleavage confirmed apoptosis induction in SH-SY5Y cells after 24 h of treatment (Figure 7) which persisted after 2 days of recovery (Figure S11). We also observed a transient increase in H2A.X phosphorylation, which had normalized after recovery (Figures 7 and S11). Strikingly, while the response of SH-SY5Y cells to omoMYC/HB*pep*-SP coacervates closely resembles the response in HEK293 cells, SK-N-BE(2) cells remain largely unaffected, with only slight increases in cleaved PARP as well as in sub-G1 cells after 24 h of treatment (Figures 7 and S12), thus closely resembling the effect in HeLa cells (Figure 3). Phalloidin staining followed by confocal microscopy showed no notable changes in the actin staining in SH-SY5Y cells exposed to omoMYC/HB*pep*-SP coacervates, and no other morphological differences between treatments for either of the two cell lines were observed (Figures 7 and S13).

**Figure 7:**
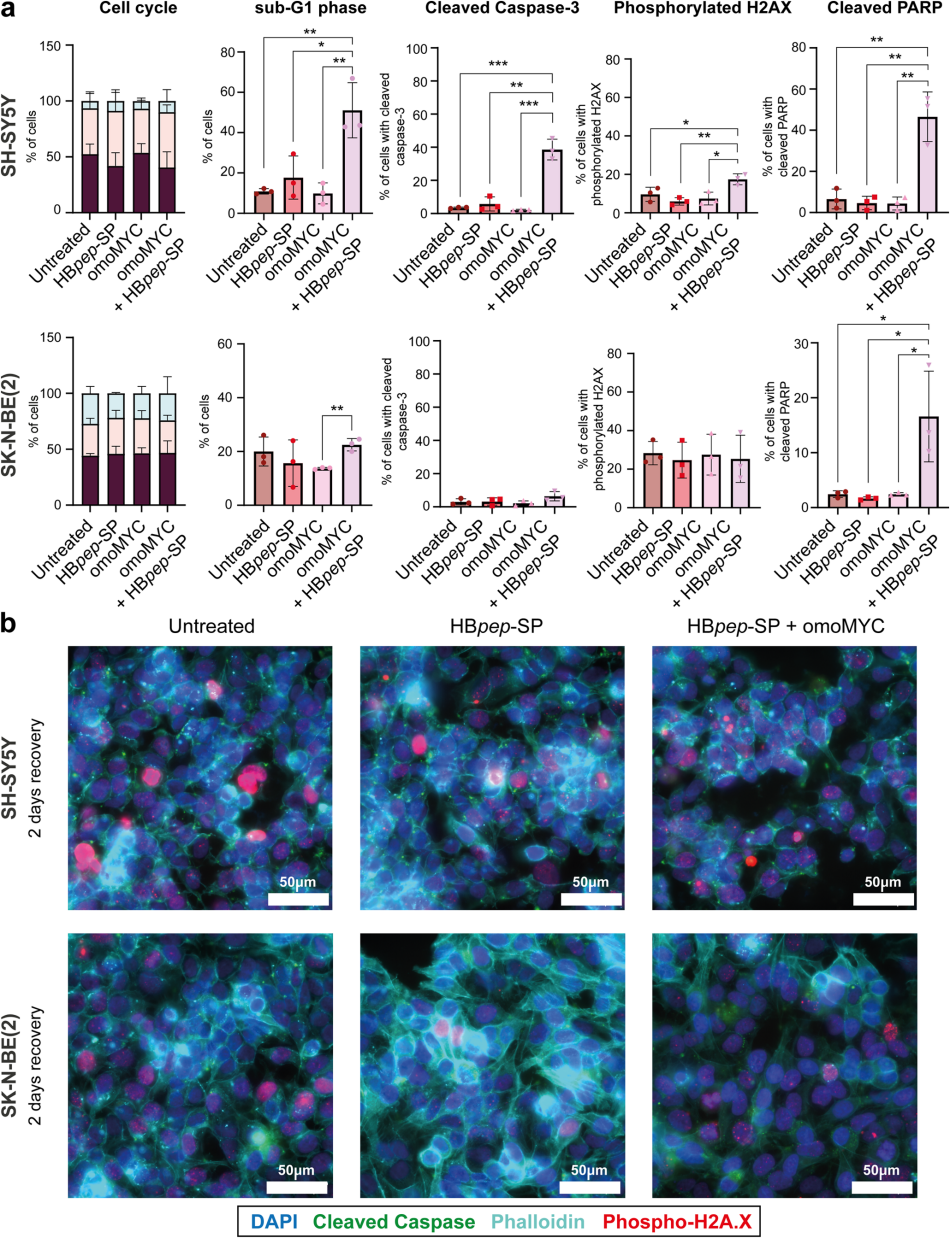
omoMYC/HB*pep*-SP -coacervate treatment activates apoptosis in SH-SY5Y but not SK-N-BE(2) cells after 24 h treatment. (a) FACS analysis of SH-SY5Y cells (top row) after 24 h treatment with omoMYC/HB*pep*-SP HB*pep*-SP coacervates. Neither omoMYC nor HB*pep*-SP alone significantly affected the cell cycle in any of the cell lines, but only a few cells remained for cell cycle analysis following the combined treatment (first column). omoMYC delivered by coacervates induced a significant increase in cell death, as indicated by the increase in sub-G1 cells and activation of caspase-3 (second and third columns) as well as increased PARP cleavage (fifth column). SK-N-BE(2) cells (bottom row) did not display any changes in response to treatment except for an increase in cleaved PARP. Data are presented as mean ± SD, *n* = 3 biological independent replicates. Statistical significance was determined by one-way ANOVA, Tukey’s multiple comparisons test, with a single pooled variance. Statistical significance: *p < 0.05, **p < 0.01, ***p < 0.001, ****p < 0.0001. (b) Confocal microscopy images of SH-SY5Y (top) and SK-N-BE(2) (bottom) cells after 24 h treatment followed by 2 days of recovery. Markers as indicated. Representative images from three biologically independent experiments are presented (*n* = 3). Images were acquired using 395/488/555/640 nm laser lines. Scale bars: 50 μm.

## Discussion

HB*pep*-SP has been shown to enable the uptake of various biomolecules, including peptides, proteins, and nucleic acids over a wide range of molecular weights.^20,21^ In this study, we demonstrate that HB*pep*-SP significantly enhances the cellular uptake of the omoMYC peptide, overcoming its intrinsic limitations in cell penetration. By delivering omoMYC into HeLa, HEK293, SH-SY5Y, and SK-N-BE(2) cells, we were able to delineate factors for omoMYC sensitivity and investigate its biological effects including mechanistic insights of action.

The ability to deliver omoMYC into cells at submicromolar treatment concentrations reveals some striking cell type specificity of its apoptotic effect. In line with our observations, Ellenbroek et al. reported HeLa cells to be less sensitive to MYC inhibition than HEK293 cells.^15^ We further find that the SH-SY5Y and SK-N-BE(2) human neuroblastoma cell lines exhibit a significant difference in omoMYC sensitivity, as judged by viability changes and increased apoptotic markers. Notably, Western blot analysis revealed that the omoMYC-sensitive cell lines in our study have low endogenous MYC levels. Importantly, Ellenbroek and co-workers used an omoMYC analogue, DuoMYC, with improved cellular uptake, which allowed treatment at similarly low inhibitor doses as facilitated by HB*pep*-SP.^15^ We speculate that with efficient coacervate delivery, low concentrations of omoMYC are sufficient to trigger apoptosis in HEK293 and SH-SY5Y cells but may not be able to overcome the strong MYC signaling in HeLa and SK-N-BE(2) cells. Importantly, our data do not exclude other causes for the difference in sensitivity. A recent study has shown that longer treatment (72 h) with higher doses of omoMYC (20 μM) reduces proliferation of the *MYCN*-amplified neuroblastoma cell lines Kelly and IMR32.^25^ Further investigations are needed to elucidate the underlying mechanism of this cell type specificity and to identify factors that predict treatment response. We anticipate that the ability of coacervates to access concentration ranges lower than those required for direct uptake will provide valuable insights.

The efficient delivery of omoMYC into different cell types furthermore enables mechanistic comparisons. The fact that H2A.X phosphorylation remains largely unchanged in all cell lines analyzed following treatment suggests that omoMYC does not directly induce DNA damage;^29^ however, replication stress may trigger a DNA damage response (DDR).^27,28,34,35^ A detailed understanding of the molecules involved in DDR could provide a rationale for synthetic lethality approaches, such as Checkpoint kinase 1 inhibition, to enhance cytotoxic effects and overcome resistance in nonresponsive cells or cancer models. The induction of caspase-3-dependent apoptosis by the coacervate-mediated delivery of omoMYC is particularly noteworthy, as evasion of apoptosis in the sympathoadrenal lineage of the neural crest is one of the hallmarks of neuroblastoma development.^36^ Targeting apoptotic signaling pathways has been explored as a promising strategy for neuroblastoma therapy. However, many neuroblastoma models rely on antiapoptotic proteins such as Bcl-2, Mcl-1, and IAP signaling, which contribute to poor prognosis and therapy resistance.^37^ The use of BH3 mimetics with combinations of antiapoptotic Bcl-2 family protein inhibitors, such as ABT-199/Venetoclax, has previously shown synergistic responses.^38–40^ Combining coacervate-mediated delivery of omoMYC with targeted therapies that disrupt antiapoptotic signaling pathways, such as BH3 mimetics or inhibitors of Bcl-2 family proteins, may offer synergistic therapeutic effects and open avenues for improved treatment outcomes, particularly for high-risk neuroblastoma patients.

## Conclusions

Here, we show that HB*pep*-SP coacervates facilitate the efficient intracellular delivery of the omoMYC peptide. We find that omoMYC/HB*pep-*SP coacervates, but not omoMYC or HB*pep-*SP alone, induce apoptosis in HEK293 as well as in SH-SY5Y cells while not showing any strong effect in HeLa or SK-N-BE(2) cells. The latter two cell lines which express high endogenous MYC levels^41^ were more resistant and recovered from the coacervate-mediated omoMYC treatment. This in turn indicates that the therapeutic effect is likely dependent on the ability of omoMYC to disrupt MYC signaling, which potentially is compromised when cellular MYC levels are very high compared to the omoMYC concentration. The possibility to study the effects of lower doses of omoMYC than required for spontaneous uptake thus allows refinement of the proposed mechanism and temporarily facilitates more specific future studies in cancer models in mice. Our findings also suggest that lower doses could reduce potential off-target effects, improve safety, and enhance specificity in MYC-driven tumors.

These findings also highlight the versatility and efficacy of HB*pep-*SP coacervates as a delivery system for bioactive peptides. Our results thus demonstrate the efficient delivery of a therapeutically viable miniprotein without the need for chemical modification, enabling investigations of its cellular activity. The direct implication of our proof-of-concept study is that the development and mechanisms of new peptide-based therapeutics can be investigated even for peptides/cargo molecules that are lacking intrinsic cell-penetrating properties.

## Supplementary Material



## Notes

Materials and methods; secondary structure analysis of omoMYC-eGFP and omoMYC (Figure S1); HB*pep*-SP coacervates enable cellular uptake of omoMYC-eGFP (Figure S2); omoMYC coacervates have no nonspecific cytotoxic effects on HEK293 and HeLa cells (Figure S3); 24 h treatment with omoMYC/HB*pep*-SP coacervates followed by 2 days of recovery induces apoptosis in HEK293 but not in HeLa cells (Figure S4); analysis of cell cycle and apoptosis in HEK293 and HeLa cells (Figure S5); analysis of apoptosis and DNA damage markers in HEK293 cells (Figure S6); analysis of apoptosis and DNA damage markers in HeLa cells (Figure S7); Western blot analysis of MYC levels (Figure S8); uptake of non-GFP-tagged omoMYC in SH-SY5Y cells (Figure S9); omoMYC coacervates do not result in nonspecific cytotoxic effects either in SH-SY5Y or SK-N-BE(2) cells (Figure S10); omoMYC-coacervate treatment activates apoptosis in SH-SY5Y and SK-N-BE(2) cells (Figure S11); analysis of cell cycle and apoptosis in SH-SY5Y and SK-N-BE(2) cells (Figure S12); and analysis of apoptosis and DNA damage markers in SH-SY5Y and SK-N-BE(2) cells (Figure S13) (PDF)
