# Supplementary material for: Engineered Peptide Coacervates Enable Efficient Intracellular Delivery of the MYC Inhibitor omoMYC
Source: Mol Pharm. 2025 Apr 30;22(6):3478–89. doi: 10.1021/acs.molpharmaceut.5c00468 (PMC12135055; doi:10.1021/acs.molpharmaceut.5c00468)
Supplement: Supplementary file 1 [file mp5c00468_si_001.pdf]

Supporting Information

Engineered peptide coacervates enable efficient intracellular delivery of the MYC inhibitor omoMYC

Carmine P. Cerrato*, Martin Krkoška*, Yue Sun, Judit Liaño-Pons, Qi Ying Neo, Thibault Vosselman, Mohammad Alzrigat, Borek Vojtěšek, David P. Lane, Marie Arsenian Henriksson#, Ali Miserez#, and Michael Landreh#

* Equal contribution

# Co-corresponding authors

Contents

Materials and Methods

Supplementary Figures 1 - 13

Materials and Methods

Chemicals and coacervates

Chemicals were purchased from Sigma unless noted otherwise. HB*pep*-SP was synthesized as described (Sun *et al*, Nature Chemistry 14:274–283, 2022).

omoMYC and omoMYC-eGFP expression and purification

The gene encoding MGHis10-omoMYC or omoMYC-eGFP-His6 were cloned into pET-26b(+) vectors. Transformation was performed into chemically competent BL21 (DE3) *E. coli* cells with the respective constructs. Cultures grown overnight were inoculated at a 1:50 ratio into Luria-Bertani (LB) medium containing 70 mg/L kanamycin or 100 mg/L carbenicillin, respectively. Cultures were grown at +37°C with continuous shaking at 200 rpm until an OD_{600} of 1 was reached. Protein expression was induced by the addition of 1 mM isopropyl-β-D-thiogalactopyranoside (IPTG) and continued overnight at +18°C with shaking at 150 rpm. Cells were harvested by centrifugation at 6,000 x g for 20 minutes at +4°C, and pellets stored at -80°C until purification. Purification was adapted from previous literature.[13] Briefly, pellets were resuspended in 50 mM Tris pH 7.5 and frozen at -20°C overnight. Lysozyme (1 mg/ml),

DNAse I (10 ug/mL), and $MgCl_2$ (2 mM) were added to the samples and incubated on ice for 1 h with occasional mixing by inverting the tubes. Samples were sonicated on ice for 5 min (0.5 sec on – 1 sec off, total sonication time 5 min), at 20% power, followed by an overnight freeze-thaw cycle at -20°C. Cell debris was removed by centrifugation at 15,000 x g for 15 min at +4°C. Both supernatant and pellet fractions were collected. The pellets were resuspended in 20 mM Tris, 20 mM imidazole, 500 mM NaCl, and centrifuged at 15,000 x g for 15 min at +4°C. The resuspension and centrifugation step were repeated three times. 10 µL of each sample (supernatant, resuspended pellet) were mixed with 10 µL of 2x Laemmli sample buffer containing DTT and heated at +95°C for 5 min. Samples were loaded onto 4-20 % SDS-PAGE gels and run 200 V for 25 min. Gels were stained with Coomassie blue and destained by washing the gel with warm water (x5) and then by adding a destaining solution of water/ethanol/acetic acid (50/40/10). The destaining solution was changed twice every 30 min and then replaced with water overnight. Gels were imaged using ChemiDoc XRS+ system (Bio-Rad, Hercules, CA, USA).

Mass spectrometry

Purified omoMYC and omoMYC-eGFP were buffer-exchanged into 100 mM ammonium acetate, pH 6.9, using BioSpin columns (BioRad, Solna, Sweden) and loaded directly into individual electrospray capillaries (Thermo Waltham, MA, USA). Mass spectra were acquired on a Micromass LCT time-of-flight mass spectrometer (MS Vision, Almere, The Netherlands) equipped with an offline nanospray source. The capillary voltage was 1.5 kV, the cone voltage 50 V, and the RF lens 1.5 kV. The pressure in the ion source was maintained at 9.0 mbar. Spectra were visualized using MassLynx 4.1 (Waters, Wilmslow, UK).

CD Spectroscopy

Purified omoMYC and omoMYC-eGFP were buffer exchanged with PBS (Thermo Fisher Scientific, city, USA) with 1 mM of 2-mercaptoethanol (Merck, Germany) using Bio-Spin Bio-Gel P-6 and P30 chromatography columns (Bio-Rad, city, USA), where the final concentrations used for analysis were 0.45 mg/ml and 0.9 mg/ml respectively. PBS with 0.1 mM of 2-mercaptoethanol was used as blank. Samples were prepared in a quartz cuvette with optical path length of 0.1 mm. Data was collected using AVIV Model 420 Circular Dichroism Spectrometer (AVIV) in

wavelength steps of 1 nm, with wavelength ranging from 180 nm to 260 nm, and an averaging time of 0.1 s over 3 scans. All CD experiments were performed at +25°C. The CD data obtained were processed on OriginPro 2024b (OriginLab Corporation, city, USA), where the data was normalized against the blank readings and averaged over three scans. Adjacent averaging smoothing of three points and Savitzky-Golay smoothing of 10 points were applied to the omoMYC and omoMYC-eGFP spectra respectively.

Cell culture and treatments

The human embryonal kidney cell line HEK-293 (ATCC, VA, USA) was cultured in Minimum Essential Medium (MEM; 41090-028, Gibco) supplemented with 10% heat-inactivated (HI) fetal bovine serum (FBS; A5670801, Gibco), 1% non-essential amino acids (NEAA; 11140050, Gibco), and 1% penicillin-streptomycin (P/S; 15070063, Gibco). Cervical adenocarcinoma HeLa cells (ATCC, VA, USA) were grown in Dulbecco's Modified Eagle Medium (DMEM; 31966-021, Gibco), 10% HI FBS and 1% P/S. The neuroblastoma cell line SH-SY5Y (ATCC, VA, USA) and SK-N-BE(2) (ATCC, VA, USA) cells were cultured in BenchStable™ DMEM/F12 (A4192001, Gibco), supplemented with 10% HI FBS, 1% NEAA, and 1% P/S. Cell line authentication was performed based on Eurofins analysis (Genomics, Ebersberg, Germany), and regular testing for mycoplasma contamination was conducted by MycoAlert™ PLUS Mycoplasma Detection Kit (LT07-318, Lonza, Basel, Switzerland). Cells were maintained in a 95% humidified atmosphere of 5% CO_2 at +37°C. Passaging was performed twice a week after exposure to PBS and trypsin/EDTA. Cell numbers were determined using a Countess 3 FL Automated Cell Counter (Invitrogen, Waltham, MA, USA). After seeding at appropriate densities to ensure that cells reached a maximum of 90% confluency by the end of the experiment, the cells were grown for 24 hours before transfection with coacervates (HB*pep*-SP) loaded with omoMYC at a concentration of 0.5 µM. The transfection was performed in OPTI-MEM media (31985070, Gibco, Waltham, MA, USA) for 4 hours in a serum-free environment, followed by incubation in complete media for up to 24 hours. Then, the media was replaced with fresh complete medium, and the cells analyzed for effects at the times indicated in the Figure legends.

omoMYC-eGFP delivery

HeLa and HEK293 cells were seeded in 35 cm^2 dishes at a density of 1×10^5 cells and cultured for 24 hours before transfection. The medium was then removed, and cells were washed twice with PBS before adding 900 µL of Opti-MEM. Freshly prepared omoMYC-eGFP-loaded coacervates (100 µL) were added, consisting of 10 µL HB*pep*-SP stock solution (10 mg/mL in 10 mM acetic acid) mixed with 90 µL omoMYC-eGFP buffer (250 µg/mL in 10 mM phosphate buffer, pH 6.5, ionic strength 100 mM fixed by NaCl). After 4 hours of incubation, cells were washed with pH 5.0 PBS twice to remove free coacervates and imaged using a fluorescence microscope (Eclipse Ti2, Nikon, Tokyo, Japan). Untreated cells or incubated with free omoMYC-eGFP or HB*pep*-SP were used as control groups.

Immunofluorescence

Cells were seeded in 18-well plates (ibidi, 81816), transfected, and grown as described above. Following 1, 3, or 7 days of cell culturing post treatment, cells were washed with 2 x PBS and fixed in 4% paraformaldehyde (PFA) in PBS for 20 min at R.T. and stored at +4°C in PBS. Permeabilization and blocking was performed with 5% goat serum, 0.25% Triton-X, and 1% BSA in PBS for 1 h. Cells were incubated with primary antibodies against phospho-Histone H2A.X (Ser139) monoclonal antibody (CR55T33) (Cat. 14-9865-82, Invitrogen, Waltham, MA, USA) at [10 µL/mL] and cleaved Caspase 3 (Cleaved Asp 175) polyclonal antibody (Cat. PA5-114687, Invitrogen, Waltham, MA, USA) at [5 µL/mL] overnight at +4°C. Following washes with 0.1% Tween-20 in PBS x 3, cells were incubated for one h with secondary antibody AlexaFluor488 F(ab')2 fragment of goat anti-rabbit IgG (H+L) (Cat. A-11070, Invitrogen, Waltham, MA, USA) at 1:200 dilution, AlexaFluor568 F(ab')2 fragment of goat anti-mouse IgG (H+L) (Cat. A-11019, Invitrogen, Waltham, MA, USA) at 1:200 dilution, and Phalloidin-iFluor 647 Reagent (Cat. A22287, Invitrogen, Waltham, MA, USA) at 1:100 dilution, and wells washed 3 x PBS. iBidi mounting medium containing DAPI (Cat. 50011, Ibidi, Gräfelfing, Germany) was added to the wells and the cells imaged on a Nikon Eclipse Ti series confocal microscope equipped with an S Plan Fluor 10x/0.3NA DIC L N1 N1 or an S PLAN ELWD 60X/0,70 objective (Nikon Melville, NY, USA), a Full Multiband Quad filter (FF01-440/521/607/700-25) and a Zyla sCMOS camera (Andor, Oxford Instruments, Belfast, UK); laser lines 395, 488, 555, and 640 nm were used to excite DAPI, AlexaFluor488, AlexaFluor568, Phalloidin-iFluor 647,

respectively. Images were analyzed using ImageJ software (NIH, Bethesda, MD, USA) or the NIS-Elements Advanced Research 5.02.03 software (Nikon, Melville, NY, USA).

omoMYC Immunofluorescence

After 24 h treatment with omoMYC or HB*pep*-SP or both peptides in combination, SH-SY5Y cells grown on glass coverslips were fixed with 4% paraformaldehyde solution for 15 min at RT, washed with PBS and incubated for 1 h with blocking solution (0.25% (v/v) Triton X-100, 3% (w/v) BSA (Merck/Sigma-Aldrich, St. Louis, MO, USA) in PBS). Next, coverslips were incubated with mouse anti-omoMYC ([21-1-3], Cat. #: 153657, CancerTools, London, UK) at a 1:200 dilution over night at +4°C in a wet chamber. After washing with PBS, the AlexaFluor 568-conjugated goat anti-mouse secondary antibody (Invitrogen, Waltham, MA, USA) was added for 1 h at RT. After washing with PBS, nuclei were stained for 5 min with DAPI (1:10,000, Invitrogen, Waltham, MA, USA) and washed again. Coverslips were mounted on slides using ProLong Diamond Antifade Mountant media (Thermo Fisher Scientific, Waltham, MA, USA). Images were taken using Z-stack with a confocal microscope Zeiss LSM700 (Oberkochen, Germany). The images were processed using ImageJ (NIH, Bethesda, MD, USA) and are representatives of two independent experiments.

Western blot analysis

Whole-cell extracts of HEK293, HeLa, SH-SY5Y, and SK-N-BE(2) were prepared using RIPA buffer (Merck/Sigma-Aldrich, St. Louis, MO, USA) supplemented with Halt Protease and Phosphatase Inhibitor Mixture (Thermo Fisher Scientific, Waltham, MA, USA). Protein samples were denatured for 5 min at +95°C in Sample Reducing Agent and Loading Buffer (Invitrogen, Waltham, MA, USA), and run in a 4-12% Bis-Tris Plus Gel (Invitrogen, Waltham, MA, USA), together with a PageRuler™ Plus Prestained protein ladder (Thermo Fisher Scientific, Waltham, MA, USA). Transfer was carried out using the Trans-Blot Turbo Nitrocellulose Transfer Packs and Turbo Transfer System (Bio-Rad, Hercules, CA, USA). Ponceau stain (Merck/Sigma-Aldrich, St. Louis, MO, USA) was used to verify even transfer and as protein loading control. Membranes were blocked for 1 h in 5% non-fat milk, followed by an incubation at +4°C overnight with a rabbit anti-pan-MYC primary antibody ([EPR18863], ab195207, Abcam, Cambridge, UK). The next day, membranes were washed and incubated for 1 h with a goat anti-rabbit horseradish peroxidase (HRP)-conjugated

secondary antibody (1:3000, Dako, P0447 and P0448, Agilent Technologies, Santa Clara, CA, USA) in 5% non-fat milk. Proteins were detected using SuperSignal West Dura Chemiluminescent Substrate (Thermo Fisher Scientific, Waltham, MA, USA) and imaged with the ChemiDoc XRS+ system (Bio-Rad, Hercules, CA, USA). Images were processed with ImageLab (Life Science Research, Bio-Rad, Hercules, CA, USA) and protein bands quantified using ImageJ (NIH, Bethesda, MD, USA). The images and quantifications shown are representatives of three independent experiments.

PrestoBlue viability assay

Cell viability was assessed using the PrestoBlue® Cell Viability Reagent (A13262, Invitrogen, Waltham, MA, USA), a cell-permeable, resazurin-based solution as colorimetric assay. Cells were seeded in 96-well plates at a density of 7,000 cells per well in 100 µL media and incubated at +37°C with 5% CO_2. After the indicated time point, 10 µL of PrestoBlue reagent was added to each well. After 10 min of incubation, the absorbance and fluorescence signals were measured on a Tecan Spark 20M multiplate reader (Männedorf, city, Switzerland) employing Tecan´s SparkControl Magellan software version 1.2., and SpectraMax i3x (Molecular Devices, San Jose, CA, USA) employing SoftMax Pro software version 7.1.2, both at λ_{ex}570 nm or λ_{ex}520 nm and λ_{em}590 nm (Tecan, Männedorf, Switzerland) at λ_{ex}570 nm or λ_{ex}520 nm and λ_{em}590 nm employing Tecan´s SparkControl Magellan software version 1.2. The cell viability experiments were performed in three biologically independent experiments with three technical replicates per experiment. Results were normalized to cells without treatment. After the measurement, the reagent was removed, and fresh growth medium was added to support continued cell proliferation for the two and six days recovery time points.

Lactate dehydrogenase (LDH) release

Cellular cytotoxicity was assessed using the CyQUANT LDH Cytotoxicity Assay kit (C20300, Invitrogen, Waltham, MA, USA) according to the manufacturer's protocol, which measures LDH release, indicating plasma membrane damage. Briefly, 50 µL of cell culture medium from each sample (10 µL of sterile, ultrapure water were added to one set of triplicate wells of cells for spontaneous LDH activity; 10 µL of 10X Lysis Buffer were added to one set of triplicate wells of cells for maximum LDH activity) were transferred to a new 96-well plate, and 50 µL of Reaction Mixture was added to each

sample well. After 30 minutes incubation at RT protected from light, the reaction was stopped by adding 50 µL of Stop Solution, and absorbance was measured at 490 nm and 680 nm using a Tecan Spark 20M multiplate reader (Männedorf, city, Switzerland), with the level of formazan formation being directly proportional to the amount of LDH released onto the medium. The LDH activity was determined by subtracting the 680 nm absorbance value (background) from the 490 nm absorbance before calculation of % Cytotoxicity. The % cytotoxicity was calculated using the following formula:

$$\% \ Cytotoxicity = \left[\frac{Compound\ treated\ LDH\ activity\ -\ Spontaneous\ LDH\ activity}{Maximum\ LDH\ activity\ -\ Spontaneous\ LDH\ activity}\right] x\ 100$$

Flow cytometry analyses (FACS)

Cells were harvested, washed with PBS, and fixed and permeabilized using the BD Cytofix/Cytoperm™ Fixation/Permeabilization Kit (#554714, BD Pharmingen, San Jose, CA, USA) on ice for 20 min. Following permeabilization, cells were washed with 1% bovine serum albumin (BSA) in PBS. For apoptosis detection, cells were stained using the FITC Active Caspase-3 Apoptosis Kit (#550480, BD Pharmingen, San Jose, CA, USA) according to the manufacturer’s instructions. For cell cycle analysis, cells were subsequently stained with FxCycle™ Violet (F10347, Invitrogen, Waltham, MA, USA) in PBS and incubated for 30 min at RT, protected from light.

In separate assays, fixed and permeabilized cells were incubated for 1 h at RT, protected from light, with PARP1 (cleaved Asp214) Monoclonal Antibody (HLNC4; #53-6668-42) and Phospho-Histone H2A.X (Ser139) Monoclonal Antibody (CR55T33; #12-9865-42, both from eBioscience, Thermo Fisher Scientific, San Diego, CA, USA). After washing steps, all samples were analyzed using a FACSVerse flow cytometer (Becton Dickinson, Franklin Lakes, NJ, USA) and FACSuite™ software (version 1.0.5, BD). At least 10,000 single cells were collected per sample, with doublets and debris excluded. The results were expressed as the percentage of cells positive for cleaved PARP1, H2A.X, or active caspase-3 related to selected cell cycle phases. Cell cycle distribution was further analyzed using ModFit LT software (version 6.0, Verity Software House, Topsham, ME, USA).

Statistical analyses

The results of at least three biological independent experiments are shown as the mean ± SD. Statistical significance was determined by Ordinary one-way ANOVA with Multiple comparisons, and multiple *t*-tests. Graphs were created using GraphPad Prism 10 (San Diego, CA, USA).

Supplementary Figures

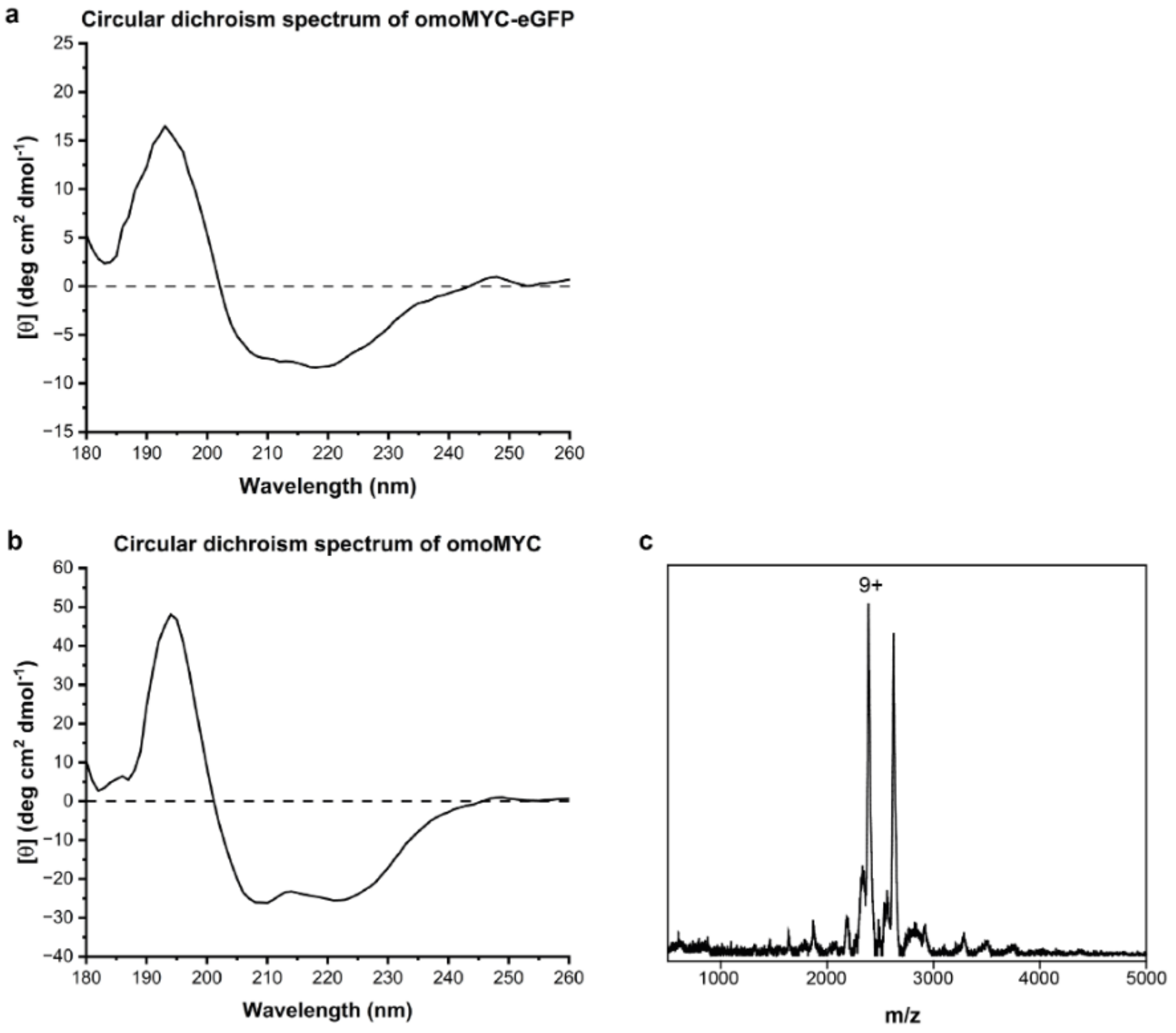

Figure S1. Secondary structure analysis of omoMYC-eGFP and omoMYC. Circular dichroism (CD) spectra of omoMYC-eGFP **(a)** show a mixed alpha-helical and random coil structure, likely due to the influence of the flexible linker and the eGFP domain; in contrast, omoMYC adopts a predominantly alpha-helical confirmation. **(b)** Both structures are consistent with their predicted structures. **(c)** Native MS analysis of omoMYC shows a covalent dimer.

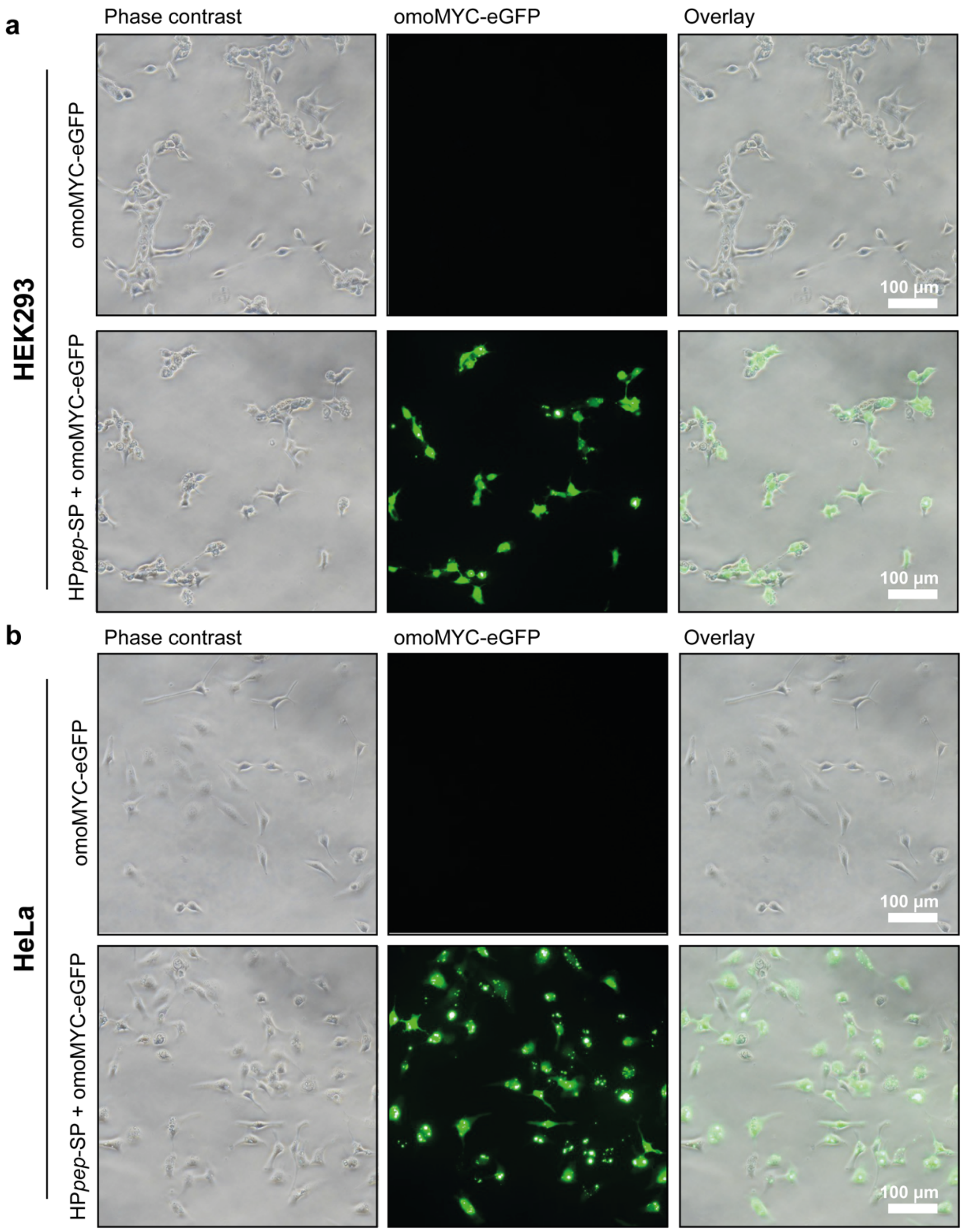

Figure S2. HB*pep*-SP coacervates enable cellular uptake of omoMYC-eGFP. (a) Fluorescence microscopy of HEK293 cells incubated for 4 h with omoMYC-eGFP at a final concentration of 0.5 µM shows no detectable uptake (top row). Under identical incubation conditions, omoMYC-eGFP/HB*pep*-SP coacervates are taken up by HEK293 cells, resulting in cytosolic GFP fluorescence (bottom row). **(b)** omoMYC-eGFP alone is not internalized into HeLa cells (top row) whereas readily internalized when delivered by HB*pep*-SP coacervates (bottom row). Representative images out of three independent biological experiments are shown (n=3). Scale bars: 100 µm.

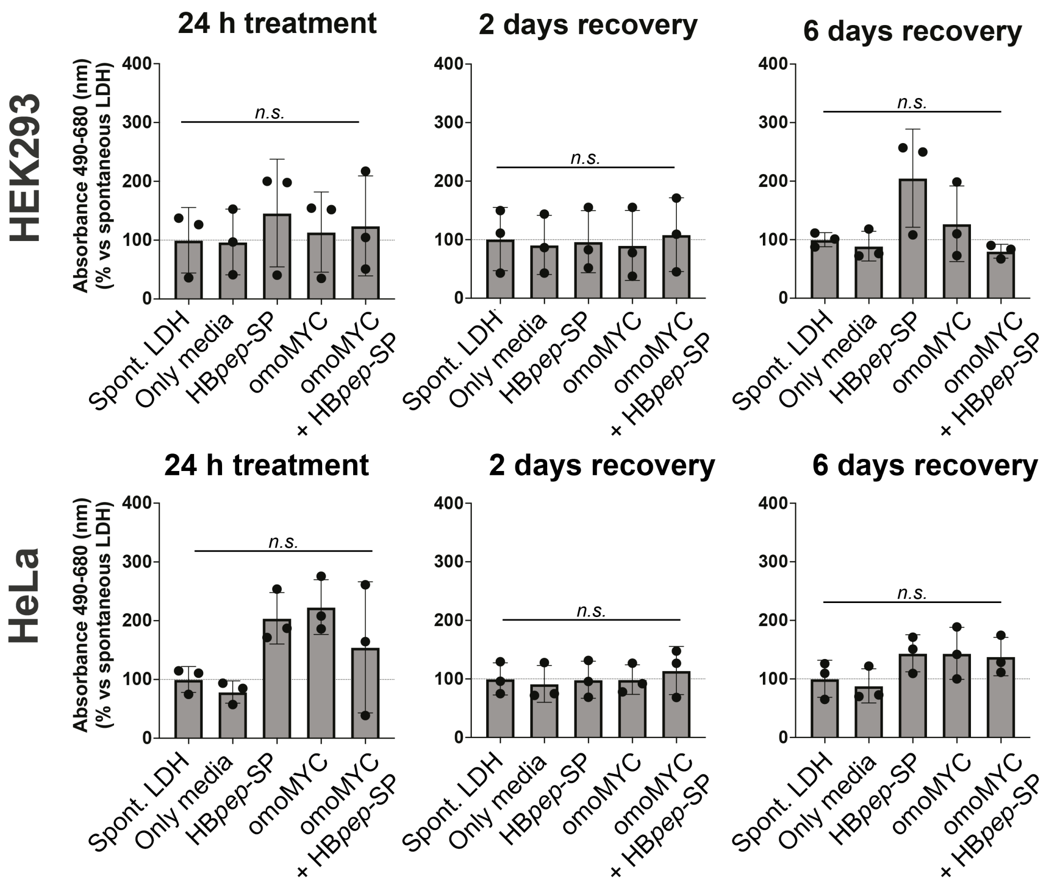

Figure S3 omoMYC coacervates have no non-specific cytotoxic effects on HEK293 and HeLa cells. Cells were analyzed with the CyQUANT LDH Cytotoxicity Assay following 24 h incubation as well as after 24 h and two or six days of recovery. At none of the time points, neither HEK293 (top row) nor HeLa (bottom row) cells show significantly increased lactate dehydrogenase (LDH) release upon omoMYC/HB*pep*-SP coacervates delivery compared to treatment with omoMYC or HB*pep*-SP alone, indicating minimal damage to the plasma membrane. “Spontaneous (Spont.) LDH” indicates background LDH activity. Data are presented as mean ± SD, n = 3 biological independent replicates. Statistics were assessed by ordinary one-way ANOVA, Tukey’s multiple comparisons test, with a single pooled variance, yielding no significant (n.s.) differences between the any of the treatments or time-points.

2 day recovery

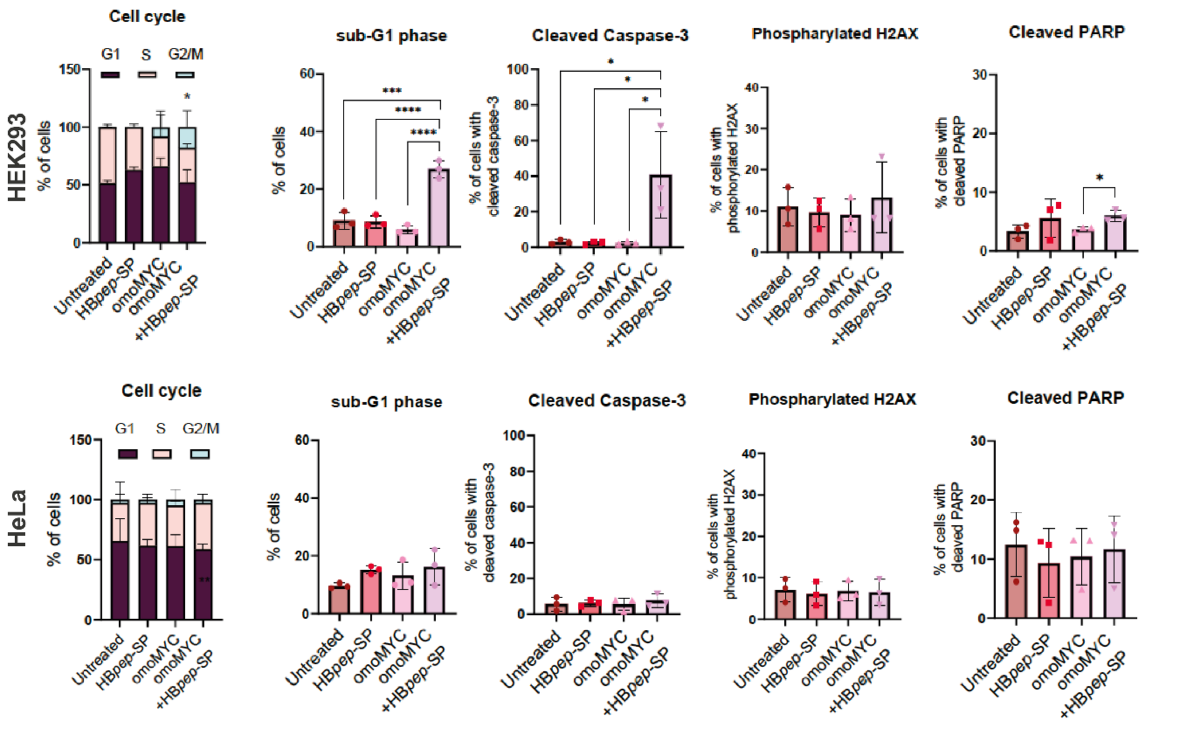

Figure S4. 24 h treatment with omoMYC/HB*pep*-SP coacervates followed by two days recovery induces apoptosis in HEK293 but not in HeLa cells. FACS analysis of cell cycle distribution and apoptosis markers in HEK293 (upper row) and HeLa (lower row) cells. 24 h treatment with omoMYC/HB*pep*-SP coacervates shows an accumulation of HEK293 cells in the G2/M phase and a reduction in S-phase whereas the were no significant cell cycle changes in HeLa cells. However, these results were confounded by a substantial sub-G1 population, suggesting potential bias and limited biological relevance. Notably, neither omoMYC nor HB*pep*-SP alone significantly affected cell cycle distribution in any cell line. While we observe increased caspase-3 as well as PARP cleavage in both cell lines, the effects are more robust in HEK293 cells. Notably, we did not detect any phosphorylated H2A.X at any of the conditions. Similarly, incubation with HB*pep*-SP or omoMYC alone did not induce any significant changes compared to untreated control cells. Mean ± SD, n=3 biological replicates. Statistical significance: * $p < 0.05$, ** $p < 0.01$, *** $p < 0.001$, **** $p < 0.0001$ (*versus* relevant control); # *versus* omoMYC alone.

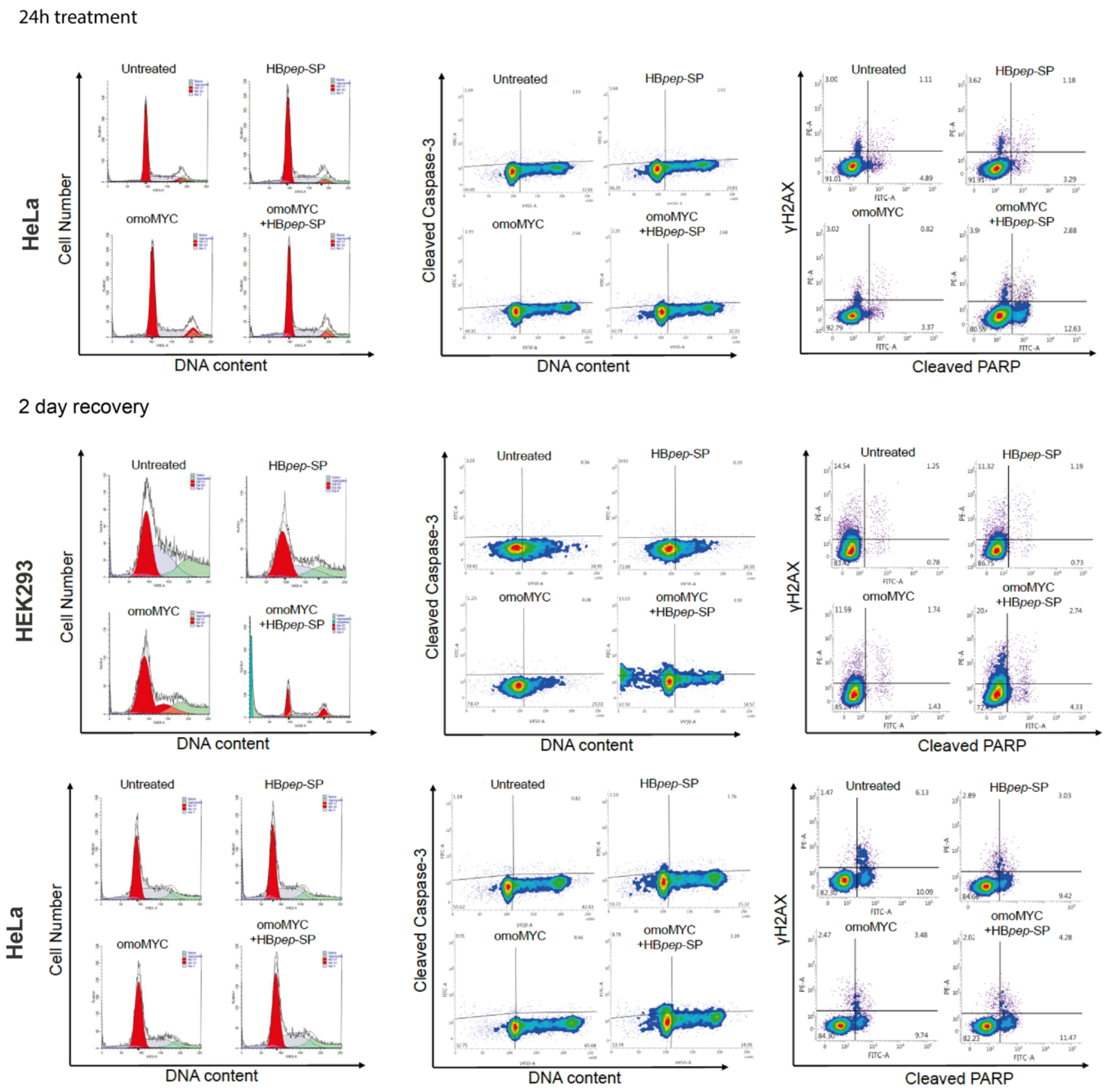

Figure S5. Analysis of cell cycle and apoptosis. Representative flow cytometry charts from three independent biological experiments quantified in Figure 3 and Figure S4, showing cell cycle analysis (left columns), cleaved caspase-3 (middle columns), and H2A.X phosphorylation (right columns) for HEK293 and HeLa cells as indicated. By two days recovery, neither HEK293 nor HeLa cells displayed standard cell cycle distribution due to high confluency, yet the combination treatment yielded insufficient numbers for a comprehensive cell cycle analysis under the same conditions.

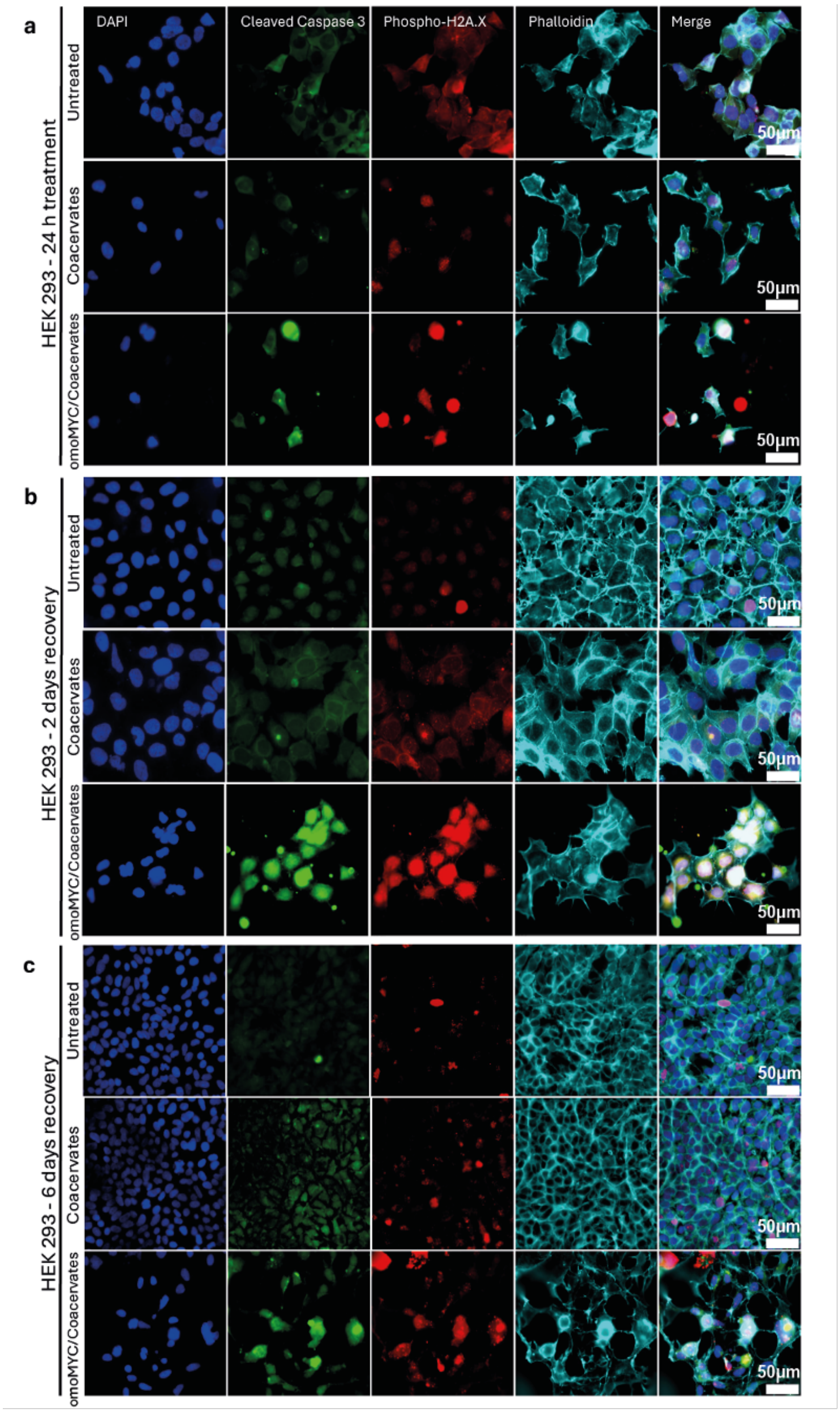

Figure S6. Analysis of apoptosis and DNA damage markers in HEK293 cells. Cells were untreated or incubated with coacervates alone or coacervates with omoMYC as indicated. Representative fluorescence images out of two independent biological experiments are shown following a 24 incubation without **(a)** as well as following **(b)** two or **(c)** six days recovery. DAPI (blue), cleaved caspase-3 (green), H2A.X phosphorylation (red), phalloidin (turquoise), and merge. Scale bars: 50 µM.

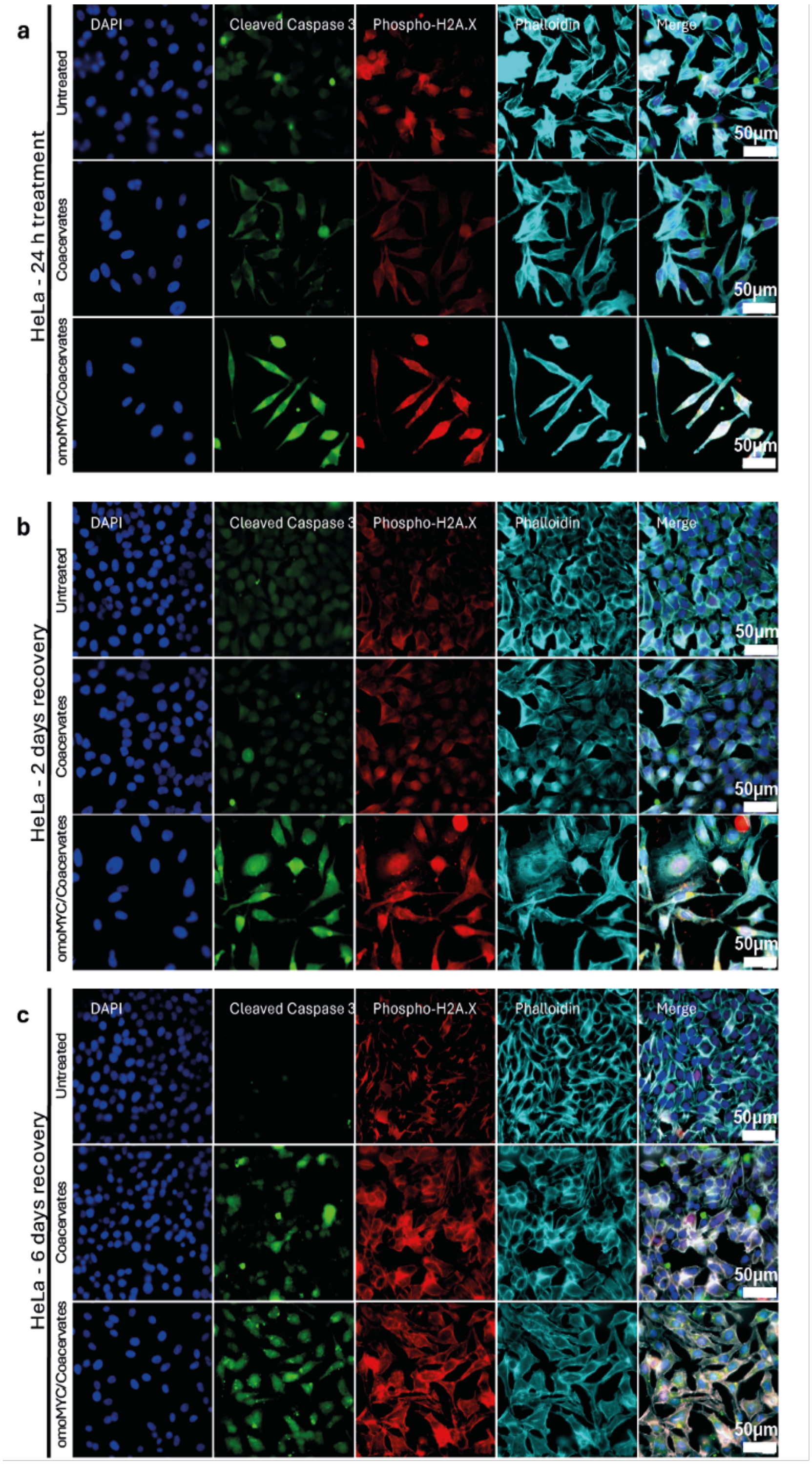

Figure S7. Analysis of apoptosis and DNA damage markers in HeLa cells. Cells were untreated or incubated with coacervates alone or coacervates with omoMYC as indicated. Representative fluorescence images out of two independent biological experiments are shown following a 24 incubation without **(a)** as well as following **(b)** two or **(c)** six days recovery. DAPI (blue), cleaved caspase-3 (green), H2A.X phosphorylation (red), phalloidin (turquoise), and merge. Scale bars: 50 µm.

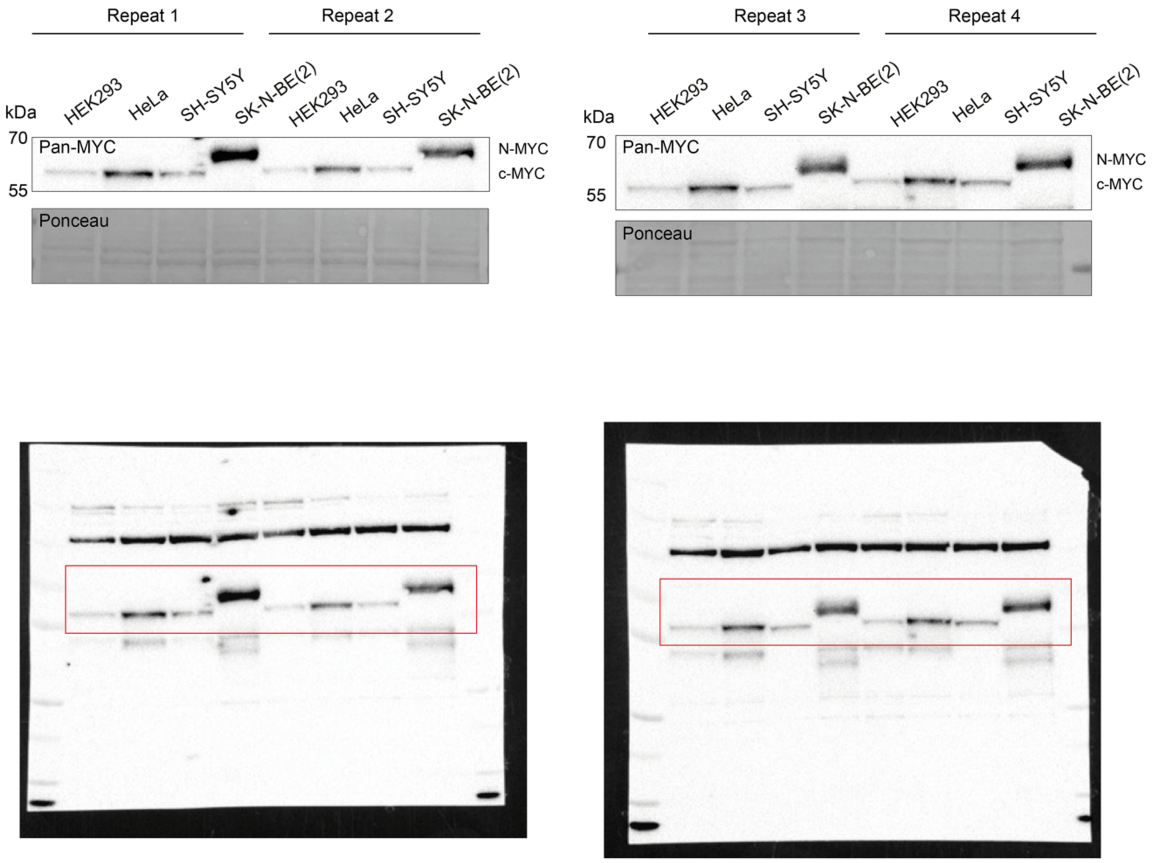

Figure S8. Western blot analysis of MYC levels. HEK293, HeLa, and SH-SY5Y cells were analyzed for c-MYC expression while SK-N-(BE(2) cells were analyzed for MYCN levels as indicated. n=4. Top row: Four individual repeats of Western blot analysis using a pan-MYC antibody (EPR18863). Ponceau was used as loading controls. Molecular weight markers in kDa are indicated to the left. Bottom row: Uncropped blots.

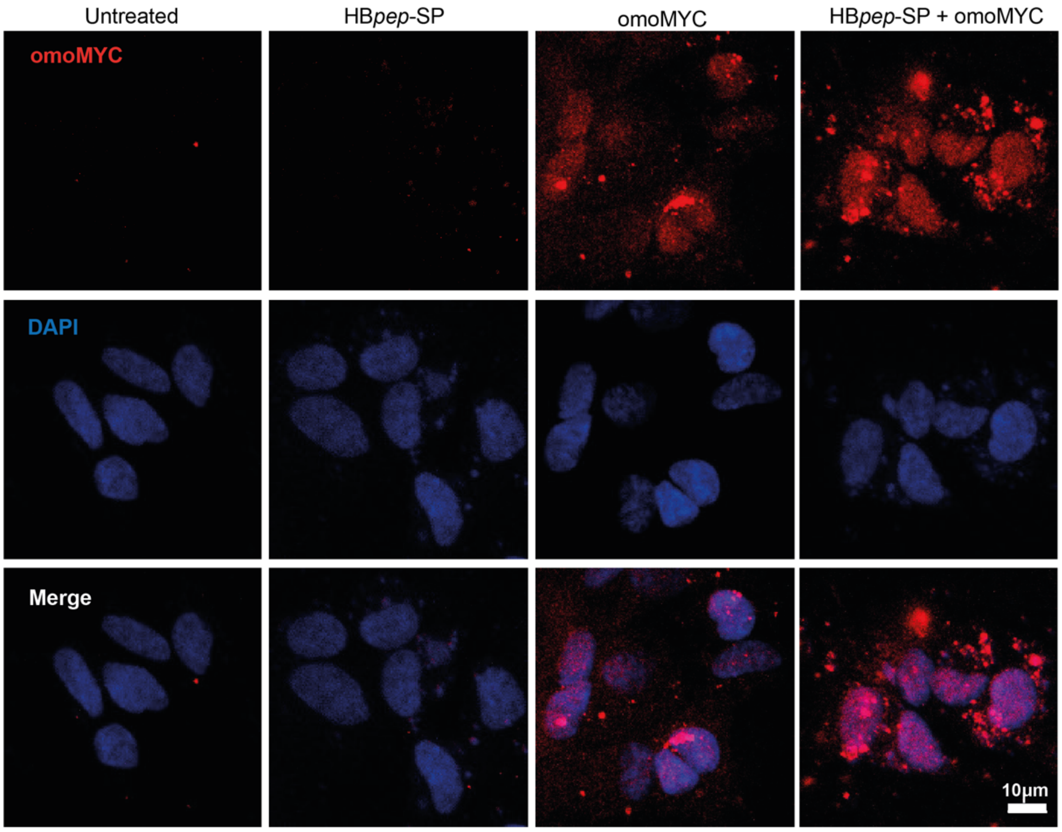

Figure S9. Uptake of non-GFP-tagged omoMYC in SH-SY5Y cells. Confocal microscopy of SH-SY5Y cells treated with 0.5 µM omoMYC with or without HB*pep*-SP stained with DAPI (blue) or the anti-omoMYC antibody with a secondary ab568 (red) show coacervate-mediated omoMYC uptake. n = 3. Scale bars: 10 µm.

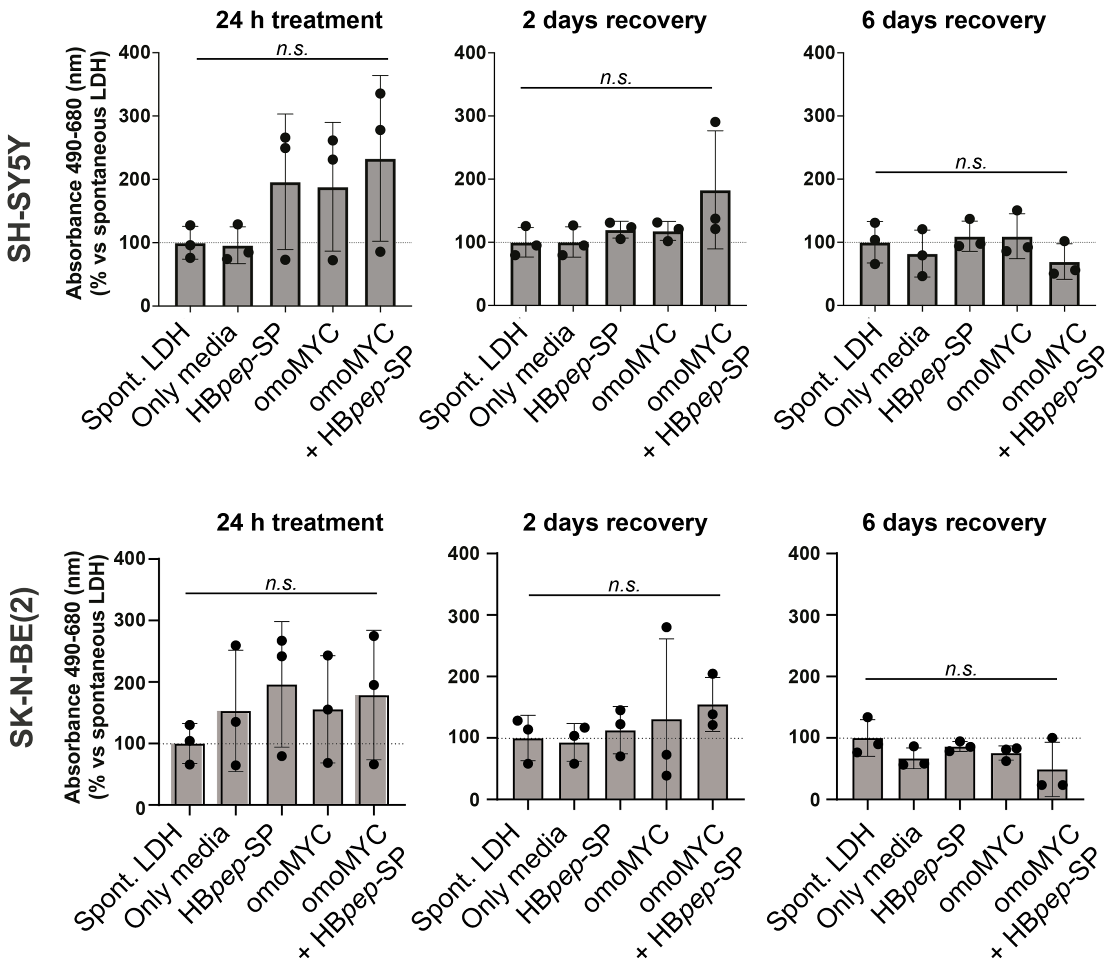

Figure S10. omoMYC coacervates do not result in non-specific cytotoxic effects either in SH-SY5Y or SK-N-BE(2) cells. Cells were analyzed with the CyQUANT LDH Cytotoxicity Assay after 24 h treatment or following a two or six day recovery as indicated. “Spontaneous (Spont.) LDH” indicates background LDH activity. Data are presented as mean ± SD, n = 3 biological independent replicates. Statistics were assessed by ordinary one-way ANOVA, Tukey’s multiple comparisons test, with a single pooled variance. No significant (n.s.) differences were observed between treatment groups at any time-point.

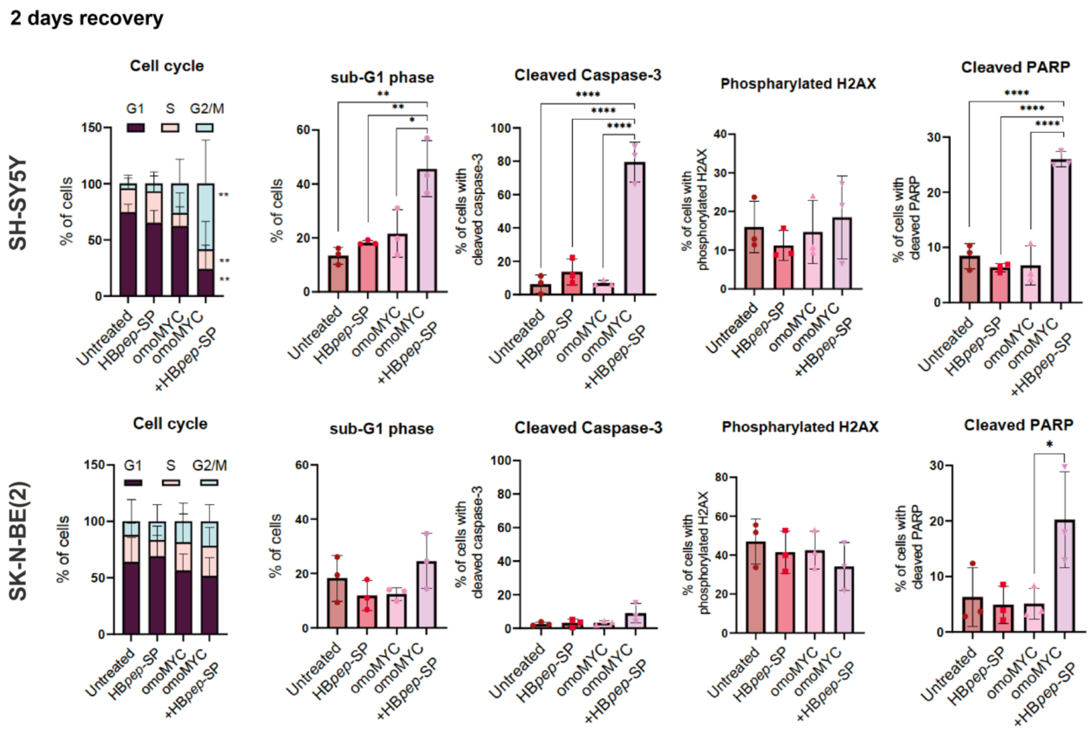

Figure S11. omoMYC-coacervate treatment activates apoptosis in SH-SY5Y and SK-N-BE(2) cells. FACS analysis of SH-SY5Y cells (top row) after 24 h treatment followed by two days recovery with omoMYC/HB*pep*-SP coacervates showed reduced G1-, S-, and accumulation in the G2/M-phase. A large sub-G1 population was detected in the combined treatment, suggesting potential bias for the cell cycle distribution. Notably, neither omoMYC nor HB*pep*-SP alone significantly affected the cell cycle in any cell line (first column). omoMYC delivered by coacervates induced a significant increase in cell death, and activation of caspase-3 (second and third columns), H2A.X phosphorylation (fourth column), as well as increased PARP cleavage (fifth column). SK-N-BE(2) cells (bottom row) did not display any changes in response to treatment except for an increase in cleaved PARP indicating certain apoptosis induction. Data are presented as mean ± SD, n = 3 biological independent replicates. Statistical significance was determined by one-way ANOVA, Tukey's multiple comparisons test, with a single pooled variance. Statistical significance: * $p < 0.05$, ** $p < 0.01$, *** $p < 0.001$, **** $p < 0.0001$.

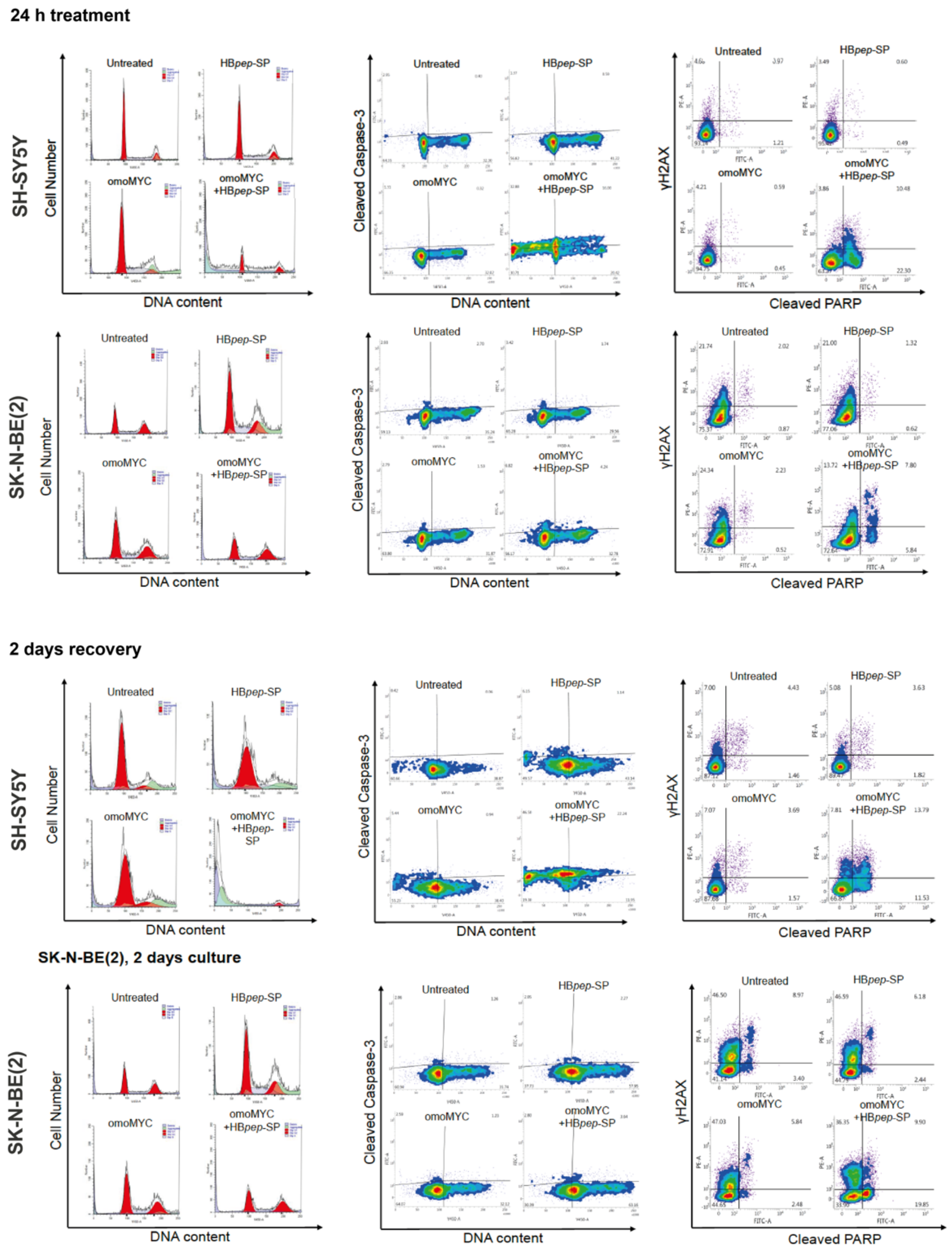

Figure S12. Analysis of cell cycle and apoptosis. SH-SY5Y (top rows) and SK-N-BE(2) (bottom rows) cells were incubated with omoMYC with or without coacervates or coacervates alone for 24 h with or without two days recovery as indicated. Cell cycle analysis (left columns), cleaved caspase-3 (middle columns), and H2A.X phosphorylation (right columns). Representative flow cytometry charts from three independent biological experiments for the data quantified in Figure 7 and Figure S11. By two days recovery, neither SH-SY5Y nor SK-N-BE(2) cells displayed standard distribution due to high confluency, yet the combination treatment yielded insufficient numbers for a comprehensive cell cycle analysis under the same conditions.

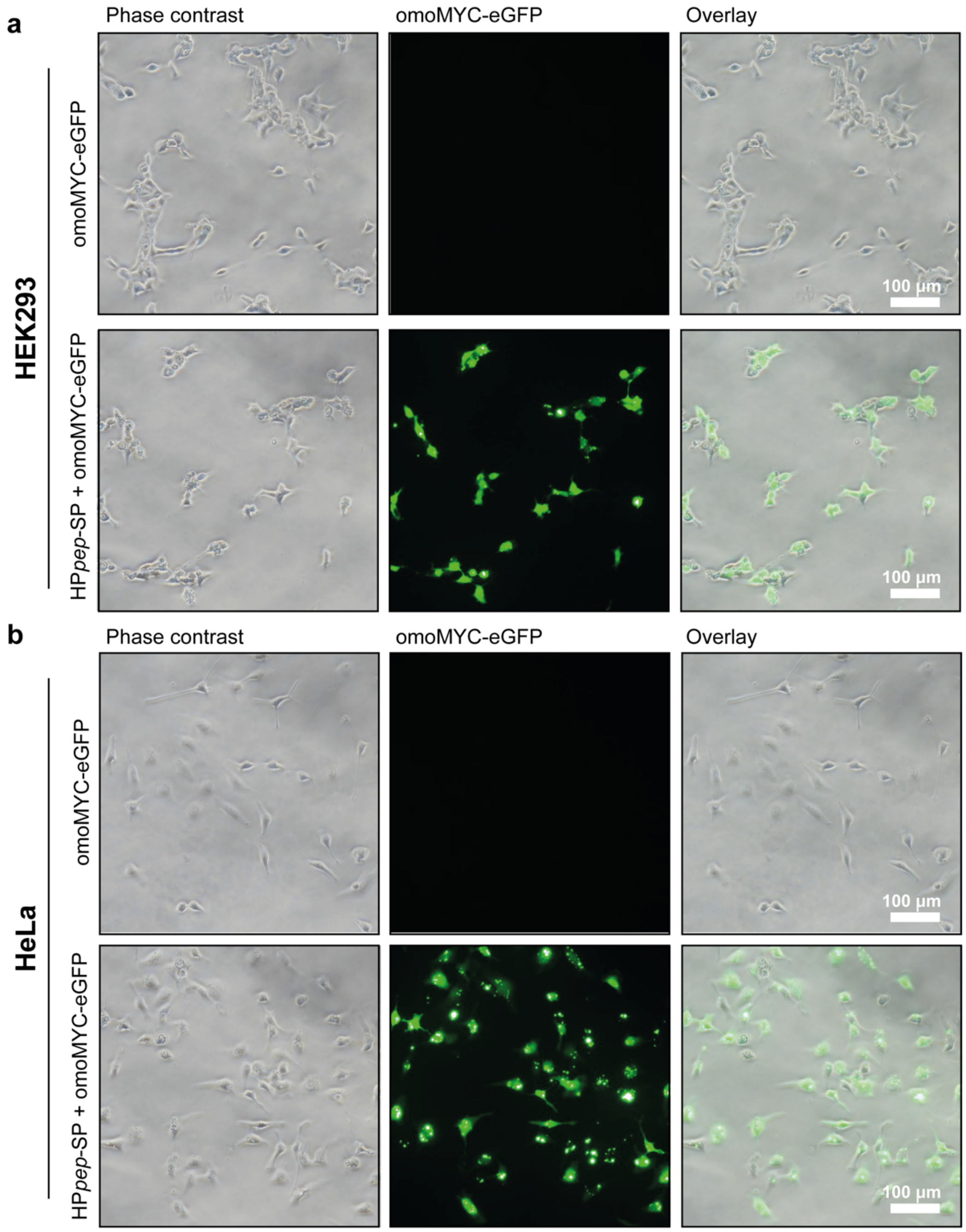

Figure S13. Analysis of apoptosis and DNA damage markers in SH-SY5Y and SK-N-BE(2) cells. SH-SY5Y (upper panels) and SKN-BE(2) (lower panels) cells were untreated or incubated with coacervates alone or coacervates with omoMYC as indicated. Representative fluorescence images from three independent biological experiments are shown for two days recovery. DAPI (blue), cleaved caspase-3 (green), H2A.X phosphorylation (red), phalloidin (turquoise), and merge. Scale bars: 25 µm.
